# Selective loss of kisspeptin signaling in oocytes causes progressive premature ovulatory failure

**DOI:** 10.1093/humrep/deab287

**Published:** 2022-01-17

**Authors:** Suvi T Ruohonen, Francisco Gaytan, Andrea Usseglio Gaudi, Inmaculada Velasco, Krisztina Kukoricza, Cecilia Perdices-Lopez, Delphine Franssen, Ipek Guler, Arfa Mehmood, Laura L Elo, Claes Ohlsson, Matti Poutanen, Manuel Tena-Sempere

**Affiliations:** 1 Research Centre for Integrative Physiology and Pharmacology, Institute of Biomedicine, University of Turku, Turku, Finland; 2 Turku Center for Disease Modeling, Turku, Finland; 3 Department of Cell Biology, Physiology and Immunology, University of Córdoba, Córdoba, Spain; 4 Instituto Maimónides de Investigación Biomédica de Córdoba and Hospital Universitario Reina Sofia, Córdoba, Spain; 5 Drug Research Doctoral Program, University of Turku, Turku, Finland; 6 Turku Bioscience Centre, University of Turku and Åbo Akademi University, Turku, Finland; 7 Centre for Bone and Arthritis Research, Institute of Medicine, Sahlgrenska Academy, University of Gothenburg, Gothenburg, Sweden; 8 CIBER Fisiopatología de la Obesidad y Nutrición, Instituto de Salud Carlos III, Córdoba, Spain

**Keywords:** oocyte, Gpr54, kisspeptins, RNA sequencing, ovulation, ovary, ovulatory failure, premature ovarian insufficiency (POI)

## Abstract

**STUDY QUESTION:**

Does direct kisspeptin signaling in the oocyte have a role in the control of follicular dynamics and ovulation?

**SUMMARY ANSWER:**

Kisspeptin signaling in the oocyte plays a relevant physiological role in the direct control of ovulation; oocyte-specific ablation of kisspeptin receptor, Gpr54, induces a state of premature ovulatory failure in mice that recapitulates some features of premature ovarian insufficiency (POI).

**WHAT IS KNOWN ALREADY:**

Kisspeptins, encoded by the *Kiss1* gene, are essential for the control of ovulation and fertility, acting primarily on hypothalamic GnRH neurons to stimulate gonadotropin secretion. However, kisspeptins and their receptor, Gpr54, are also expressed in the ovary of different mammalian species, including humans, where their physiological roles remain contentious and poorly characterized.

**STUDY DESIGN, SIZE, DURATION:**

A novel mouse line with conditional ablation of Gpr54 in oocytes, named OoGpr54^−/−^, was generated and studied in terms of follicular and ovulatory dynamics at different age-points of postnatal maturation. A total of 59 OoGpr54^−/−^ mice and 47 corresponding controls were analyzed. In addition, direct RNA sequencing was applied to ovarian samples from 8 OoGpr54^−/−^ and 7 control mice at 6 months of age, and gonadotropin priming for ovulatory induction was conducted in mice (N = 7) from both genotypes.

**PARTICIPANTS/MATERIALS, SETTING, METHODS:**

Oocyte-selective ablation of *Gpr54* in the oocyte was achieved *in vivo* by crossing a Gdf9-driven Cre-expressing transgenic mouse line with a Gpr54 LoxP mouse line. The resulting OoGpr54^−/−^ mouse line was subjected to phenotypic, histological, hormonal and molecular analyses at different age-points of postnatal maturation (Day 45, and 2, 4, 6 and 10–11 months of age), in order to characterize the timing of puberty, ovarian follicular dynamics and ovulation, with particular attention to identification of features reminiscent of POI. The molecular signature of ovaries from OoGpr54^−/−^ mice was defined by direct RNA sequencing. Ovulatory responses to gonadotropin priming were also assessed in OoGpr54^−/−^ mice.

**MAIN RESULTS AND THE ROLE OF CHANCE:**

Oocyte-specific ablation of *Gpr54* caused premature ovulatory failure, with some POI-like features. OoGpr54^−/−^ mice had preserved puberty onset, without signs of hypogonadism. However, already at 2 months of age, 40% of OoGpr54^−/−^ females showed histological features reminiscent of ovarian failure and anovulation. Penetrance of the phenotype progressed with age, with >80% and 100% of OoGpr54^−/−^ females displaying complete ovulatory failure by 6- and 10 months, respectively. This occurred despite unaltered hypothalamic *Gpr54* expression and gonadotropin levels. Yet, OoGpr54^−/−^ mice had decreased sex steroid levels. While the RNA signature of OoGpr54^−/−^ ovaries was dominated by the anovulatory state, oocyte-specific ablation of Gpr54 significantly up- or downregulated of a set of 21 genes, including those encoding pituitary adenylate cyclase-activating polypeptide, Wnt-10B, matrix-metalloprotease-12, vitamin A-related factors and calcium-activated chloride channel-2, which might contribute to the POI-like state. Notably, the anovulatory state of young OoGpr54^−/−^ mice could be rescued by gonadotropin priming.

**LARGE SCALE DATA:**

N/A.

**LIMITATIONS, REASONS FOR CAUTION:**

Conditional ablation of Gpr54 in oocytes unambiguously caused premature ovulatory failure in mice; yet, the ultimate molecular mechanisms for such state of POI can be only inferred on the basis of RNAseq data and need further elucidation, since some of the molecular changes observed in OoGpr54^−/−^ ovaries were secondary to the anovulatory state. Direct translation of mouse findings to human disease should be made with caution since, despite the conserved expression of *Kiss1*/kisspeptin and *Gpr54* in rodents and humans, our mouse model does not recapitulate all features of common forms of POI.

**WIDER IMPLICATIONS OF THE FINDINGS:**

Deregulation of kisspeptin signaling in the oocyte might be an underlying, and previously unnoticed, cause for some forms of POI in women.

**STUDY FUNDING/COMPETING INTEREST(S):**

This work was primarily supported by a grant to M.P. and M.T.-S. from the FiDiPro (*Finnish Distinguished Professor*) Program of the Academy of Finland. Additional financial support came from grant BFU2017-83934-P (M.T.-S.; Ministerio de Economía y Competitividad, Spain; co-funded with EU funds/FEDER Program), research funds from the IVIRMA International Award in Reproductive Medicine (M.T.-S.), and EFSD Albert Renold Fellowship Programme (S.T.R.). The authors have no conflicts of interest to declare in relation to the contents of this work.

**TRIAL REGISTRATION NUMBER:**

N/A.

## Introduction

Kisspeptins have taken a central stage in reproductive physiology and medicine. Initially regarded as metastasis-suppressors (Ohtaki *et al.*, 2001), kisspeptins, encoded by the *Kiss1* gene, and their receptor, Gpr54 (aka, Kiss1r), are now recognized as indispensable components of the neuroendocrine circuits controlling puberty and reproduction (Oakley *et al.*, 2009; Pinilla *et al.*, 2012). Inactivating mutations of *Gpr54* or *Kiss1* genes result in central hypogonadism, with impuberism and infertility, both in humans and rodents (Seminara *et al.*, 2003; d'Anglemont de Tassigny *et al.*, 2007; Topaloglu *et al.*, 2012). The primary site for the reproductive actions of kisspeptins is the hypothalamus, where discrete populations of Kiss1 neurons, located in the arcuate nucleus (ARC) and the rostral periventricular area, are found in multiple species (Garcia-Galiano *et al.*, 2012a), and kisspeptins potently stimulate GnRH secretion, thereby eliciting gonadotropin secretion to drive gonadal maturation and function (Oakley *et al.*, 2009; Pinilla *et al.*, 2012).

The fundamental brain effects of kisspeptins do not preclude the possibility of additional peripheral actions in the control of reproduction. Expression of *Kiss1* and *Gpr54* has been documented at other levels of the hypothalamic–pituitary–gonadal axis, including the testis and ovary (Castellano *et al.*, 2006; Salehi *et al.*, 2015; Hu *et al.*, 2017); yet, the physiological relevance of kisspeptin signaling in the gonads is still the matter of debate. Direct kisspeptin actions on GnRH neurons seem sufficient to complete puberty and attain fertility (Kirilov *et al.*, 2013; Leon *et al.*, 2016), whereas global *Gpr54* null mice and humans can be forced to ovulate, if appropriately primed with gonadotropins (Seminara *et al.*, 2003; Gaytan *et al.*, 2014). However, subtle alterations and premature aging of the reproductive axis occur in rodents devoid of Gpr54 signaling elsewhere than in GnRH neurons (Leon *et al.*, 2016), while ovulatory rescue in *Gpr54* knock-out (KO) mice is quantitatively incomplete and requires intensive priming (Gaytan *et al.*, 2014). Undoubtedly, the prominent central (GnRH-dependent) effects of kisspeptins dominate their reproductive actions, masking the potential peripheral effects of kisspeptins, which nonetheless may contribute to the precise modulation and fine tuning of gonadal function. However, the nature and (patho)physiological relevance of such gonadal actions of kisspeptins are yet to be fully disclosed.

Ovarian expression of the components of the Kiss1 system has been documented in multiple species. While some discrepancy on their actual cellular distribution within the ovary is found in the literature, the expression of *Kiss1*/kisspeptins and Gpr54 has been reported in the porcine, bovine, rodent (rat, mouse, hamster) and primate ovary (Castellano *et al.*, 2006; Hu *et al.*, 2017); the latter, including marmoset monkeys and humans (Gaytan *et al.*, 2009). The physiological roles of local kisspeptin signaling in the ovary are yet to be fully defined, but may involve a variety of functions, from the control of steroidogenesis (Owens *et al.*, 2018) and puberty onset (Ricu *et al.*, 2012), to ovarian aging (Hu *et al.*, 2017). Interestingly, *Kiss1* mRNA expression in the rat ovary has been shown to fluctuate according to the stage of the cycle, with peak levels at the pre-ovulatory phase; a phenomenon that is driven by the ovulatory surge of gonadotropins (Castellano *et al.*, 2006). Moreover, inhibition of prostaglandin synthesis, which is known to severely perturb ovulation, caused a marked drop of ovarian *Kiss1* mRNA levels and prevented the capacity of ovulatory doses of hCG to induce *Kiss1* expression in the rat ovary (Gaytan *et al.*, 2009). Collectively, these data indirectly pointed out a putative role of local kisspeptins in the control of ovulation. In line with this hypothesis, blockade of local kisspeptin signaling by intra-ovarian infusion of a kisspeptin antagonist reduced the number of corpora lutea (CL), as marker of ovulation, while direct ovarian injection of kisspeptin caused the opposite effect (Fernandois *et al.*, 2016). Studies in humans and other species have demonstrated that systemic injection of kisspeptins promotes egg maturation and ovulation (Matsui *et al.*, 2004; Caraty *et al.*, 2007; Jayasena *et al.*, 2014), and clinical studies supported the effectiveness of kisspeptins to induce oocyte maturation in patients with high risk of ovarian hyperstimulation syndrome (Abbara *et al.*, 2017). Yet, the fact that parenteral administration of kisspeptin evokes also potent gonadotropin responses makes impossible to tease apart local versus central actions for such ovulatory effects of kisspeptins.

In this context, experimental evidence from our group has suggested that ovarian kisspeptin signaling may play a role in follicle survival and, eventually, ovarian aging (Dorfman *et al.*, 2014; Gaytan *et al.*, 2014). Thus, *Gpr54* haplo-insufficiency, which suppressed *Gpr54* mRNA levels in the ovary, but preserved gonadotropin secretion, evoked late-onset ovarian failure, with anovulation by 11–12 months of age (Gaytan *et al.*, 2014). On the other hand, signaling via the neurotrophin receptor, NTRK2, in the oocyte seems to require preserved kisspeptin signaling to promote oocyte survival and prevent premature ovulatory failure (Dorfman *et al.*, 2014). This evidence, together with the demonstration of expression of *Gpr54* in the oocyte in rodent, canine and porcine species (Saadeldin *et al.*, 2012; Dorfman *et al.*, 2014; Cielesh *et al.*, 2017), suggested that direct kisspeptins actions in the oocyte may play a relevant role in the control of ovulation, whose failure would lead to early ovulatory failure, reminiscent of premature ovarian insufficiency (POI). However, the evidence so far available was indirect and inconclusive to unambiguously discriminate direct versus indirect oocyte actions of kisspeptins. We report here that selective elimination of *Gpr54* from the oocyte leads to a progressive POI-like condition, defined by preserved resting and early growing follicle pools, but enhanced atresia of large antral follicles and premature anovulation.

## Materials and methods

### Generation of OoGpr54^−/−^ mouse line

An oocyte-specific iCre expressing transgenic mouse line (Tg(Gdf9-icre)5092Coo/J) was obtained from Jackson Lab (stock number 011062, www.jax.org). A Gpr54-LoxP mouse line, thoroughly validated by our group previously (Garcia-Galiano *et al.*, 2012b), was transferred to the Central Animal Laboratory of University of Turku, maintained on a homozygous background, and crossed with the heterozygous Gdf9-iCre line. The crossing scheme is described in Supplementary Fig. S1A. Genotyping was conducted by PCR analyses on isolated genomic DNA from ear punches. Primer sequences and PCR conditions are listed in Supplementary Table SI. Two genotypes were generated and subsequently analyzed: iCre^−/−^::Gpr54^+/+^ (control, hereafter Cre-LoxP^+/+^) and iCre^+/−^::Gpr54^−/−^ (oocyte-specific KO, OoGpr54^−/−^). Mice were housed with same-sex littermates on a 12 h light-dark cycle with *ad libitum* access to tap water and Soy-free SDS-RM3 chow (Special Diets Services Inc., Essex, UK).

### Experimental design

#### General procedures

Only female mice were studied. Mice were euthanized at diestrus phase of the cycle by CO_2_ inhalation followed by blood aspiration via cardiac puncture and cervical dislocation. Serum was separated by centrifugation and stored at −70°C. Tissues were collected and either snap-frozen in liquid nitrogen and stored at −70°C (DNA/RNA isolation and PCR analyses), or fixed in Bouin’s Solution (ovaries) or 10% v/v buffered formalin, and embedded in paraffin for histology.

### Verification of DNA recombination and deletion of Gpr54 expression in oocytes

Genomic DNA from the ovary, uterus, hypothalamus, heart, tail, adrenal gland, kidney, liver and spleen was isolated, and genotyped by PCR analysis with primers located outside the two LoxP sites (Supplementary Fig. S1A). PCR primers and conditions are shown in Supplementary Table SI. To show the suppression of *Gpr54* expression in targeted cells, qPCR analyses of whole ovaries and isolated oocytes were conducted with hypothalamus and gonadal fat tissue used as controls. Oocytes were collected from oviducts after gonadotropin priming. For this, we used 2.5 IU pregnant mare’s serum gonadotrophin (PMSG) i.p. followed by 5 IU hCG i.p. after 47 h (n = 7–9 mice per group). Oocytes were collected after 24 h from the last injection. RNA was isolated using a Direct-zol RNA MicroPrep Kit (ovaries, oocytes; Zymo Research, CA, USA), or a trizol reagent (hypothalamus, fat; Trisure^®^, Bioline, Tauton, MA, USA). One µg of RNA was reverse-transcribed to cDNA (SensiFast cDNA synthesis kit, Bioline, London, UK) and quantified with the SYBR Green reagent (DyNAmo™ SYBR^®^ Green, Thermo Scientific) in a CFX96 real-time PCR detection system (Bio-Rad, Hercules, CA, USA). Ribosomal protein L19 was used as control in the ΔΔCt method. Primer sequences and reaction conditions are listed in Supplementary Table SI.

### Evaluation of the onset of puberty and estrous cycles

Vaginal opening was monitored daily starting at post-natal day (PND) 25 until observed opening (n = 10–12/group). Thereafter, daily vaginal smears were collected to analyze the presence of first estrus. Smear samples were stained as previously described (Hakkarainen *et al.*, 2015). Starting at 2 months of age, smear samples were collected daily for 21 consecutive days to analyze the cycle lengths.

### Ovarian histology

Groups of mice were sacrificed at the age of PND45, and 2, 4, 6 and 10 months of age. Group sizes per each genotype and age-point were as follows. Control, Cre-LoxP^+/+^ mice: n = 8, 6, 16, 8 and 9 per age group; OoGpr54^−/−^ mice: n = 8, 5, 27, 11 and 8 per age group, respectively. Bouin-fixed ovaries were embedded in paraffin, serially sectioned (7 µm-thick), and stained with hematoxylin and eosin. The ovulatory status was established in each ovary by assessing the presence of CL. In addition, to analyze whether the ovarian follicle reserve was altered or not, the number of resting follicles per ovary was counted in a randomly selected subset of mice of both genotypes, at 2, 4 and 6 months of age, with the following group sizes: Control, Cre-LoxP^+/+^ mice: n = 5, 6, 7 per age group; OoGpr54^−/−^ mice: n = 5, 8, 7 per age group, respectively. Resting follicles correspond to primordial (showing flattened granulosa cells), transitional (showing a mixture of flattened and cuboidal granulosa cells) and early primary (showing cuboidal granulosa cells but not enlarged oocyte and measuring less than 30 µm in diameter) follicles, according to previous studies (Gaytan *et al.*, 2015). To analyze the initial activation of follicle growth, the number of early growing follicles, corresponding to primary follicles containing one to two layers of cuboidal granulosa cells, enlarged oocyte and measuring between 30 and 70 µm in diameter, per ovary were counted. Follicle counts were performed through a systematic random procedure in every tenth ovarian section (starting by a randomly selected section out of the first 10 sections), and the final result was multiplied by 10 to obtain the total number of follicles per ovary. Follicles were counted in the section containing the oocyte nucleolus (for resting follicles) or the oocyte nucleus (for early grown follicles) to avoid double counting. Final follicle growth/maturation was evaluated by counting the number of mid/large antral follicles, measuring from 250 to >400 µm, taken together as large antral follicles. These follicles undergo rapid growth from estrus to proestrus, reaching pre-ovulatory size (Gaytan *et al.*, 2017). The number of large antral follicles per ovary, either healthy or atretic, were obtained by screening of all sections and counted in the section containing the oocyte nucleolus. Of note, antral follicles were considered as atretic when showing apoptotic (i) granulosa cells (defined by five or more pyknotic cells in the follicle section containing the oocyte nucleus), (ii) granulosa cell debris in the antrum and/or (iii) areas of thinning and general deformation of the granulosa layer, as signs of early atresia, together with (iv) alterations of the oocyte, at very advanced stages of atresia, in line with previous criteria (Visser *et al.*, 2007; Uslu *et al.*, 2017). Further details about the morphometric features used to define follicular atresia in ovaries from OoGrp54^−/−^ mice can be found in Supplementary Fig. S2. In addition, the presence and number of CL were also recorded.

### Serum levels of sex steroids, gonadotropins and anti-Mullerian hormone

Serum estrone (E1), estradiol (E2), progesterone (P) and testosterone (T), as well as LH and FSH levels were measured in the diestrus phase. Samples were collected between 09:00 and 12:00 h. LH was also assayed in proestrus (14:00 h) without sacrificing the animals. Due to the limited volume of serum obtained at proestrus, hormonal measurements at this phase were limited to LH. Diestrus gonadotropins were assayed at 4, 6 and 10 months of age, whereas sex steroids at diestrus and LH at proestrus were analyzed at 4 months of age. Anti-Mullerian hormone (AMH) levels were measured at diestrus from 4- and 10-month-old mice. Gonadotropins were measured by time-resolved immune-fluorometric assays as previously described (Haavisto *et al.*, 1993; van Casteren *et al.*, 2000), or with an ultrasensitive LH assay (Steyn *et al.*, 2013). Sex steroids were assayed using a previously validated method, based on gas chromatography tandem mass spectrometry (Nilsson *et al.*, 2015). AMH concentrations were determined using an ELISA Kit from AnshLabs (Catalog #AL-113), whose sensitivity was 0.33 ng/µl. Quadratic regression curve-fit was used to interpolate concentrations from optical density values of standard curve (range: 0.33–21 ng/µl).

### Ovarian transcriptomic analyses

RNAseq analyses (Illumina HiSeq 3000 sequencing) were carried out in highly-pure RNA samples from ovaries of 6-month-old control (n = 7) and OoGpr54^−/−^ (n = 8; 6 non-ovulatory and 2 ovulatory) mice at the Finnish DNA Microarray and Sequencing Centre (Turku Centre for Biotechnology, University of Turku) according to the manufacturer’s protocols (Illumina, Cambridge, UK). Raw reads were quality controlled using the FastQC tool, and the reads were aligned to the mouse reference genome version mm10 available at Illumina iGenomes (http://support.illumina.com/sequencing/sequencing_software/igenome.html) using the default settings of Tophat software v.2.0.10. Gene-wise read counts were normalized using the TMM normalization method of the edgeR R/Bioconductor package. For statistical testing, the data were further transformed using the voom approach in the limma package. Statistical comparison was performed for those genes that had expression above 0.125 in at least two samples across the data set.

### Ovarian response to external gonadotropin priming

Ovaries from control and OoGpr54^−/−^ mice (n = 7 per genotype), collected at 24 h after above described gonadotropin priming, were formalin-fixed, embedded in paraffin, and serially sectioned (7 µm-thick) and hematoxylin & eosin stained. The number and characteristics of the CL were determined according to previous criteria (Gaytan *et al.*, 2017), to discriminate the status (i.e. cycling or anovulatory) of each OoGpr54^−/−^ mouse before gonadotropin priming, as anovulatory mice showed only one generation of newly formed (hCG-induced) CL, whereas previously cycling animals show one or two additional populations of regressing CL from previous cycles. The number of oocytes in the oviduct, which matches the number of newly formed CL, was also counted. Serum AMH levels were also measured at the time of ovarian collection (i.e. 24 h after gonadotropin priming), as described in previous sections.

### Statistics

Statistical analyses were conducted using Prism software (GraphPad Prism 8.0, GraphPad Software, La Jolla, CA, USA). All data are presented as mean ± SEM. Unpaired Student’s *t*-test and one- or two-way ANOVA, followed by Sidak’s multiple comparisons test in case of significant interaction, were used to assess variation between experimental groups. The significance level was set at *P* ≤ 0.05.

A non-supervised analysis of global expression was performed using the principal component analysis and two-dimensional hierarchical clustering. Kyoto Encyclopedia of Genes and Genomes (KEGG) database was used for enrichment analysis. DAVID Bioinformatics Resources version 6.8 was used to identify KEGG pathway (Huang da *et al.*, 2009). Fisher’s exact test is used to measure the gene-enrichment in annotation terms. For multiple hypothesis testing correction, standard false Discovery rate control methods were used and the combined score calculated by multiplying logarithmic transformation of *P*-value and -score for deviation from expected rank.

### Study approval

The experimental protocol was approved by the National Animal Experiment Board of Finland, and all experiments were performed according to ICLAS guidelines for animal experimentation.

## Results

### A conditional oocyte-specific Gpr54 knock-out mouse model

A novel oocyte-specific Gpr54 KO mouse line, termed OoGpr54^−/−^, was produced by crossing the Gdf9-Cre and the Gpr54^loxP/loxP^ mouse lines (Supplementary Fig. S1A). Generation of OoGpr54^−/−^ mice was verified by a genomic DNA PCR, where a positive signal of Cre-mediated recombination was obtained only from OoGpr54^−/−^ ovaries, and not in any other tissue studied (Supplementary Fig. S1B). In addition, qPCR assays denoted a 50% reduction in *Gpr54* expression levels in the whole ovary, whereas the expression was reduced by >75% in isolated oocytes, obtained after gonadotropin-induced ovulation, in young adult OoGpr54^−/−^ mice (Supplementary Fig. S1C). These qPCR data validate our mouse model of conditional suppression of *Gpr54*, as they document a predominant reduction of *Gpr54* expression in oocytes, whereas non-oocyte sources would be responsible for the remaining *Gpr54* signal in whole ovaries of OoGpr54^−/−^ mice. In contrast, *Gpr54* mRNA levels were not suppressed in gonadal fat, and were even higher in the hypothalamus, suggesting a potential compensatory reaction in the brain to the lack of Kiss1 signaling in the ovary.

### Normal puberty onset in OoGpr54^−/−^ females

Eight OoGpr54^−/−^ mice were analyzed for pubertal onset. In seven individuals, vaginal opening, as external sign of puberty, took place at the same mean age as in control mice (Supplementary Fig. S3A), without differences in the age of the first estrus, as sign of first ovulation (Supplementary Fig. S3A). Similar onset of puberty was also verified in histological sections collected at PND45, as OoGpr54^−/−^ and control animals displayed a similar stage of ovarian maturation and predicted date of first ovulation, based on the combined analysis of their stage of follicular and corpus luteum (CL) maturation, and total numbers of fresh CL per ovary, as defined by a blinded expert analyst (Supplementary Fig. S3B–I). Notably, in one of the eight OoGpr54^−/−^ mice studied for puberty (12.5%), no sign of ovulation was detected upon histological analyses of the ovaries (Supplementary Fig. S3E–G).

### Premature ovulatory failure and abnormal cyclicity in OoGpr54^−/−^ mice

**Figure 1.**
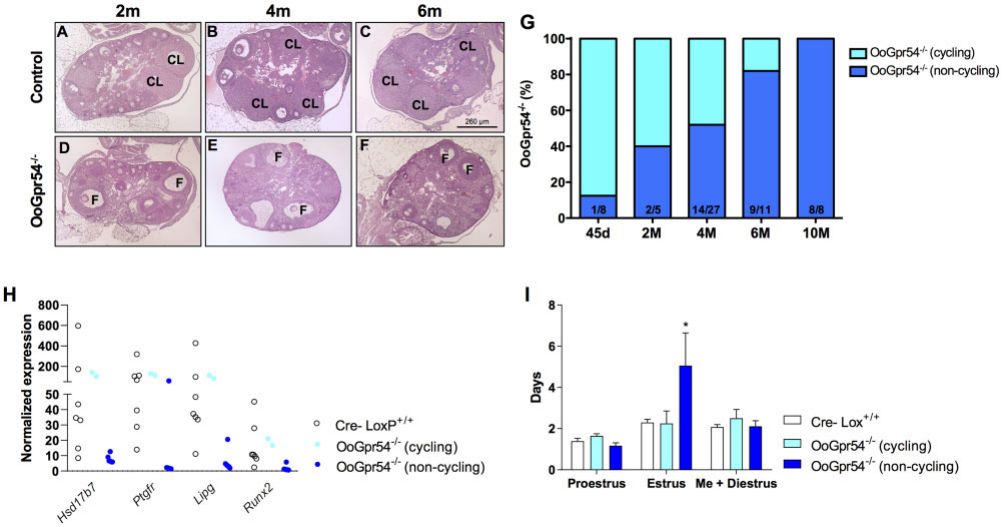
**Progressive premature ovulatory failure in OoGpr54^−/−^ mice.** In panels (**A–F**), representative hematoxylin and eosin staining of control (Cre-LoxP^+/+^) and OoGpr54^−/−^ ovaries at 2, 4 and 6 months (M) of age. Corpora lutea (CL) indicating previous ovulations are numerous in control mice, whereas there are individuals in the OoGpr54^−/−^ genotype that lack CL completely, but do have apparently healthy antral follicles (F). In panel (**G**), percentage of individuals in OoGpr54^−/−^ females per age group that do not show CL in ovaries, and therefore are considered as non-cycling. In panel (**H**), differentially expressed transcripts known to be related to the function and development of CL, providing further evidence of anovulatory phenotype in OoGpr54^−/−^ females. Data were obtained from RNAseq analysis of ovaries from 6-month-old animals. In panel (**I**), average lengths of different phases of the estrous cycle analyzed from vaginal smear samples. Values are means ± SEM, n = 7–9/group. *P < 0.05 versus control Cre-LoxP^+/+^ mice.

Already at the age of 2 months, the histology of the OoGpr54^−/−^ ovaries showed a dimorphic phenotype: 2 out of 5 (40%) KO mice showed distinct histological features reminiscent of ovarian failure characterized by an absence of CL (Fig. 1A–G). Yet, macroscopic evaluation of gonadal tissues showed no gross differences between genotypes (Supplementary Fig. S1D). Analogous observations in terms of CL and presence of follicles were also made at 4 and 6 months of age (Fig. 1A–G). Penetrance of this anovulatory phenotype progressed with age: 55% of OoGpr54^−/−^ females at 4 months, and 80% at 6 months. By 10 months of age, 100% of OoGpr54^−/−^ mice displayed features of complete ovulatory failure (Fig. 1G). Notably, at 10–11 months of age, while some control mice were yet ovulating, there were individuals showing abnormalities, such as absence of fresh CL or presence of follicular cysts in the ovaries (Supplementary Fig. S4), suggestive of an incipient compromise of the reproductive capacity. The absence of CL in 6-month-old OoGpr54^−/−^ females was further verified with transcriptomics analysis of the ovaries, which showed reduced expression levels of genes produced by the luteinized cells of the CL, selectively in the non-ovulatory animals (Fig. 1H). In contrast, the OoGpr54^−/−^ females that retained ovulatory capacity at that age were closer to controls in the expression levels of such luteinizing markers (Fig. 1H). Vaginal smear samples collected from adult females showed a similar dimorphic pattern regarding the estrus length in OoGpr54^−/−^ mice (Fig. 1I). The animals that showed an elongated estrus phase, had no CL and did not ovulate. OoGpr54^−/−^ mice with normal cyclicity displayed CL similar to controls.

### Increased follicular atresia in large follicles from OoGpr54^−/−^ ovaries

**Figure 2.**
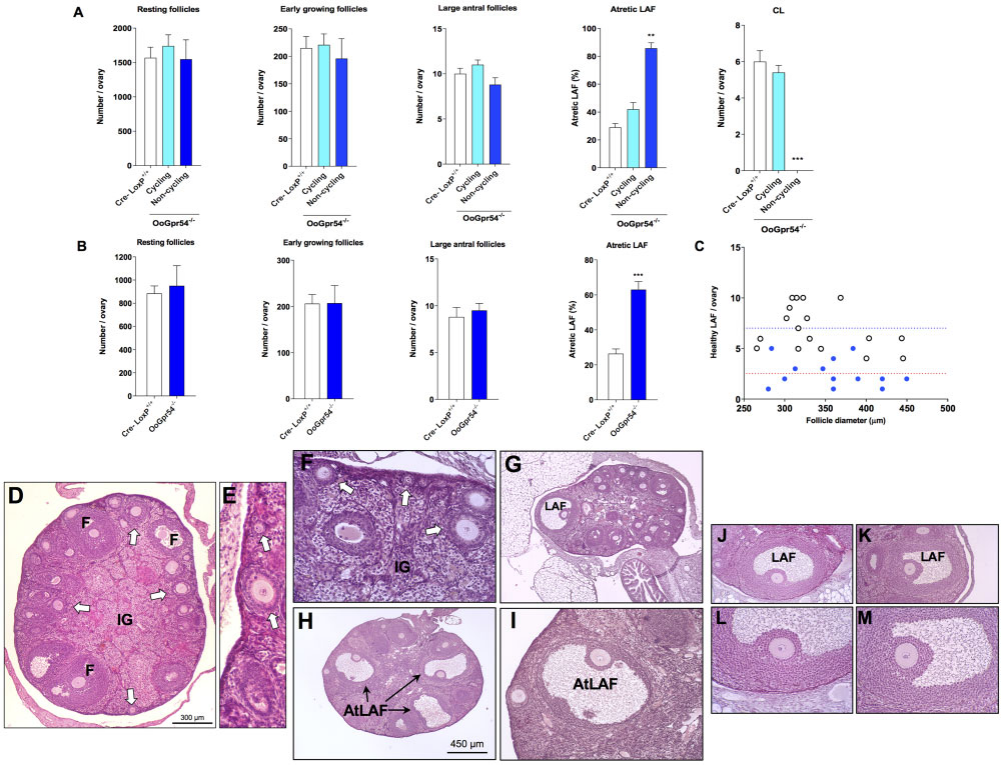
**Higher degree of follicular atresia and lack of corpora lutea in OoGpr54^−/−^ ovaries.** In panels (**A** and **B**), average number of resting, early growing, large antral and atretic large antral follicles, and corpora lutea (CL) per ovary are shown from 2 to 3-month-old (**A**) and 6-month-old (**B**) control and OoGpr54^−/−^ mice. In 2- to 3-month-old mice, follicles and CL were first quantified from all OoGpr54^−/−^ animals, after which they were divided into cycling and non-cycling based on the presence of CL. Data from the 6-month-old group are only presented from the non-cycling knock-outs, because only two animals displayed signs of ovulation. Values are means ± SEM. **P < 0.01 and ***P < 0.001 with one-way ANOVA (in A); or ***P < 0.001 with Student’s *t*-test (in B). In panel (**C**), dot-plot representation is shown of the mean number of healthy large antral follicles (>250 µm in diameter) per ovary, plotted against the mean follicle size, in control (white dots, n = 17) and anovulatory OoGpr54^−/−^ (blue dots, n = 13) mice, over the age-range of 2–6 months. In controls, the distribution of follicle size is dependent on the stage of the cycle; OoGpr54^−/−^ mice showed a similar size distribution, though the number of follicles per ovary was lower and ovulation was compromised in some individuals. In lower panels (**D**–**M**), representative photomicrographs of follicles, at various maturations states, from anovulatory OoGpr54^−/−^ ovaries are presented. Early growing (*white arrows*) and antral (*F*) follicles and interstitial gland (*IG*) were abundant (**D–F**), whereas corpora lutea were absent. Large antral follicles (*LAF*, as seen in **G**) were numerous, although many of them displayed histological signs of atresia (*AtLAF* in **H**, **I**). However, some apparently healthy LAF reached preovulatory size (**K**; at greater magnification in **M**), with morphological features identical to those in control mice (an example presented for comparative purposes in **J**; at greater magnification in **L**).

The number of resting and early growing follicles did not differ between the genotypes at 4 or 6 months of age (Fig. 2A and B). Likewise, no significant changes were detected in the absolute amount of large antral follicles between control and OoGpr54^−/−^ mice. However, a significant increase in the number of large follicles displaying clear histological signs of atresia was noticed in OoGpr54^−/−^ females. In 4-month-old mice, where the split phenotype of ovulatory failure was evident, a 3-fold increase in follicular atresia was detected only in animals not showing ovulation (Fig. 2A). In contrast, only a modest, non-significant rise was detected in ovulatory OoGpr54^−/−^ females at 4 months of age. At 6 months of age, the anovulatory phenotype was clearly dominant, with 80% of mice displaying signs of anovulation. Hence, counting of atretic follicles was only conducted in those anovulatory OoGpr54^−/−^ mice, which confirmed the highly significant rise of the percentage of large antral follicles undergoing atresia, without changes in the total number of antral follicles per ovary (Fig. 2B). Despite this observation, our histological analyses documented that even large, morphologically healthy follicles could be found in 6-month-old OoGpr54^−/−^ mice displaying an anovulatory phenotype, albeit their total number was much lower than in the corresponding controls (Fig. 2C). Representative images of follicles, at various maturations states, from anovulatory OoGpr54^−/−^ ovaries are presented in Fig. 2D–M.

### Normal gonadotropic function but suppressed ovarian secretion in OoGpr54^−/−^ mice

**Figure 3.**
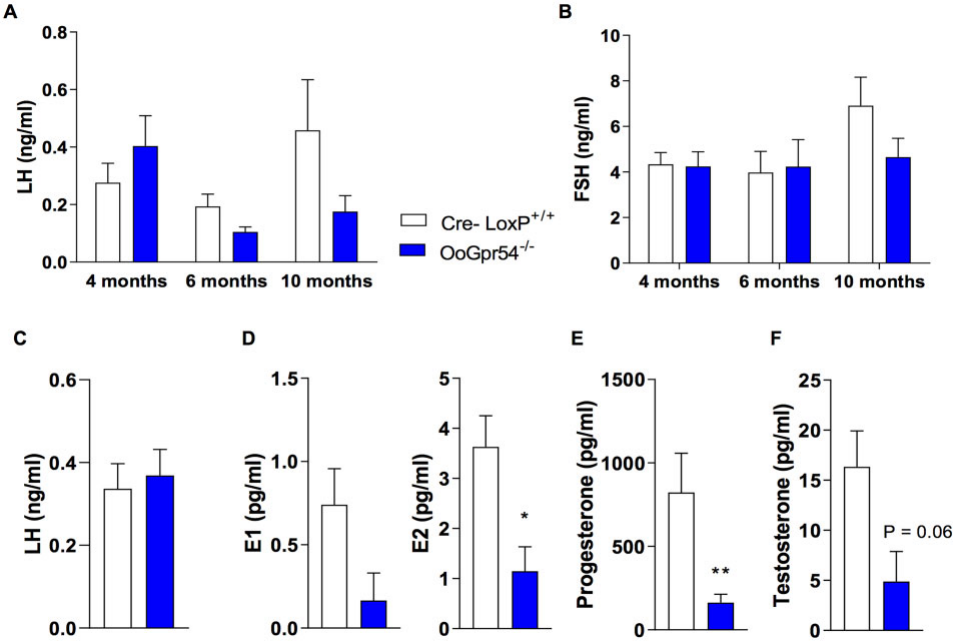
**Normal gonadotropin production but reduced sex steroid levels in OoGpr54^−/−^ mice.** Circulating LH (**A**) and FSH (**B**) concentrations at various ages, in control (Cre-LoxP^+/+^) and OoGpr54^−/−^ female mice in the morning of diestrus. In panel (**C**), serum LH levels in 4-month-old control and OoGpr54^−/−^ females at the afternoon of proestrus. In addition, the circulating levels of estrone (E1) and estradiol (E2; **D**), progesterone (**E**) and testosterone (**F**) in 4-month-old Cre-LoxP^+/+^ and OoGpr54^−/−^ females (at diestrus) are shown. Values are means ± SEM, n = 5–10/group. *P < 0.05 and **P < 0.01 with Student’s *t*-test.

Serum gonadotropin (LH and FSH) levels were measured at different ages, and found to be normal in OoGpr54^−/−^ mice (Fig. 3A and B). Analyses were performed at diestrus, since the animals were euthanized at this stage. However, as LH peaks at late proestrus, LH levels were also assayed from samples collected in the early afternoon of proestrus; i.e. before the LH surge occurs. No difference was noticed at this stage either compared to controls (Fig. 3C). Thus, central-driven regulation of the gonadal function seems to be preserved in OoGpr54^−/−^ females. In addition, AMH levels, as surrogate marker of the follicular pool (Dumont *et al.*, 2015), were measured at diestrus and found to be grossly similar between control and OoGpr54^−/−^ mice at 4- and 10 months of age, in keeping with the histological ovarian substrate of our POI-like model, in which preantral and small antral follicles, as major source of AMH (Durlinger *et al.*, 2002), are not affected. Yet, at 10 months of age, circulating AMH levels were lower than at 4 months, irrespective of the genotype, and a non-significant trend for decreased AMH in oocyte-specific Gpr54 null animals was observed (Supplementary Fig. S5). Circulating sex steroids were also determined at diestrus in 4-month-old animals. In support of the ovarian insufficiency, serum levels of estradiol and progesterone were significantly lower in OoGpr54^−/−^ mice, and a strong tendency was also observed in estrone and testosterone concentrations (Fig. 3E–G). Postmortem ovarian histology revealed that out of six animals sampled for sex steroid measurements, only one retained ovulation. For the sake of consistency, this was not included in graph analyses; yet, while progesterone levels were in the normal range in this ovulatory OoGpr54^−/−^ female, estradiol levels were nearly undetectable (*data not shown*), suggesting an incipient ovarian failure despite as yet preserved ovulation.

### Genotype- and phenotype-driven changes in the ovarian transcriptome of OoGpr54^−/−^ mice

Direct RNA sequencing was applied to identify changes in the ovarian transcriptome of OoGpr54^−/−^ mice at 6 months of age. Eight KO mice were subjected to analyses, of which two were ovulatory; seven control, Cre-/LoxP^+/+^mice, without ablation of *Gpr54* in the oocyte, served as controls. Unbiased analyses of differentially expressed transcripts followed by clustering analyses revealed a dominant role of phenotype-driven changes in the molecular signature of OoGpr54^−/−^ ovaries; i.e. changes caused by the anovulatory phenotype, which results in the absence of CL, and hence profoundly alters the relative abundance of RNA transcripts, irrespective of the absence of *Gpr54* in oocytes (Supplementary Fig. S6A). Prominent examples of such luteinizing-dependent transcripts are presented in Fig. 1C. On this basis, specific comparison of transcriptomic profiles was conducted between control and OoGpr54^−/−^ mice not displaying ovulation, namely, those showing POI-like state (N = 6). These statistical analyses revealed 161 genes that were differentially expressed (either significantly up- or downregulated) in the ovaries of non-ovulatory OoGpr54^−/−^ mice (Supplementary Table SII). Clustering based on this gene list permitted a perfect segregation between control and OoGpr54^−/−^ mice (Supplementary Fig. S6B), which was further confirmed by principal component analyses (PCA; Supplementary Fig. S6C). A fold-change diagram, representing the upregulated (47) and downregulated (114) genes in anovulatory OoGpr54^−/−^ mice, is also shown (Supplementary Fig. S7). Based on this set of differentially expressed genes, KEGG pathway enrichment analyses were applied. These analyses allowed identification of putatively upregulated pathways, including glutathione metabolism, AGE-RAGE (namely, advanced end-glycation products and their receptor) and PI3K/Akt signaling, peroxisome physiology and cancer-related pathways, in the ovaries of anovulatory OoGpr54^−/−^ mice, in which other KEGG pathways were also putatively downregulated, e.g. lysosomal and phagosome pathways, cytochrome P450-related xenobiotic metabolism pathways, peroxisome proliferator-activated receptor (PPAR) signaling pathways, amino acid degradation systems and complement cascade, among others (Supplementary Fig. S8).

**Figure 4.**
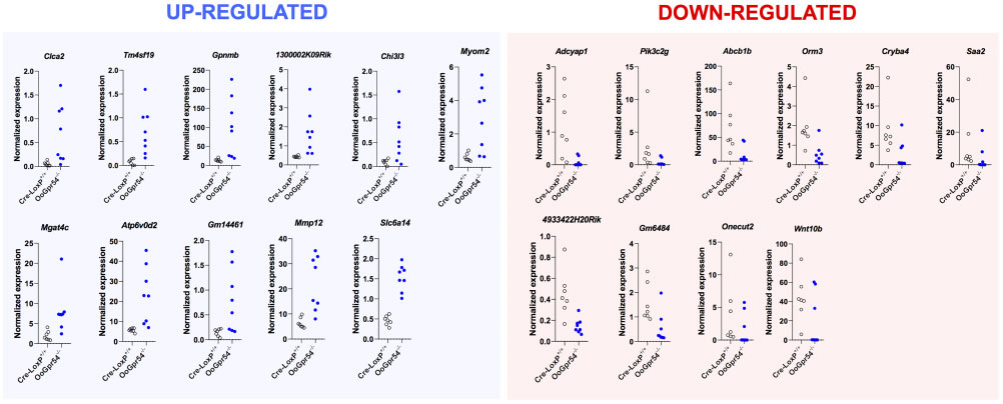
**RNAseq analysis of ovaries from OoGpr54^−/−^ mice.** Identification of the most significantly (*P* < 0.05) upregulated (*left panels*; shadowed in blue) and downregulated (*left panels*; shadowed in red) transcripts in the ovaries of OoGpr54^−/−^ mice versus control (Cre-LoxP^+/+^) females. Ovaries were collected in the morning of diestrus at 6 months of age. Individual values are plotted, n = 7–8/group.

In spite of the above, genotype-driven changes in the transcriptome profiles were also observed. Hence, statistical analyses of changes in transcript levels between the whole set of control and OoGpr54^−/−^ mice, allowed identification of a subset of 21 genes that were significantly upregulated (n = 11) or downregulated (n = 10) in the ovaries of OoGpr54^−/−^ mice, irrespective of their ovulatory state. The individual profiles of each of these transcripts are presented in Fig. 4.

### Ovulatory failure of OoGpr54^−/−^ mice can be rescued by gonadotropin priming

**Figure 5.**
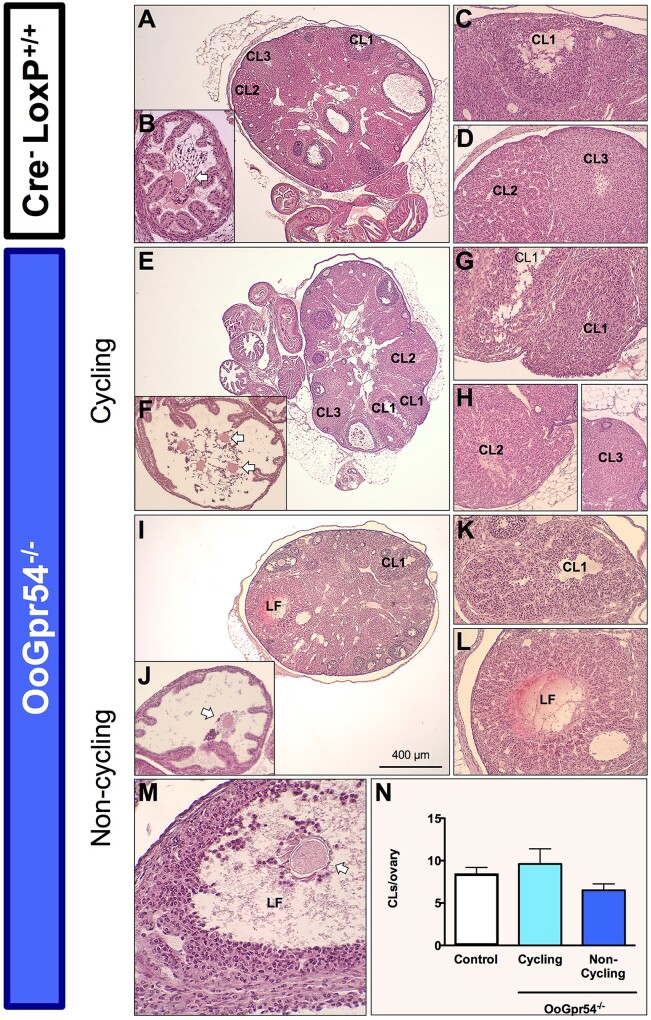
**Induction of ovulation in non-cycling OoGpr54^−/−^ mice following gonadotropin priming.** Representative H&E micrographs of ovarian responses to gonadotropin priming in 4-month-old control (Cre-LoxP^+/+^) and OoGpr54^−/−^ mice. In the upper panel, histological analysis of control mice evidenced the presence of newly formed corpora lutea (*CL1* in **A**, **C**), corpora lutea of previous cycles (*CL2*, *CL3* in **A**, **D**), and cumulus–oocyte complexes in the oviduct (*arrow* in **B**), as index of ovulation within 24-h of priming. In both cycling and non-cycling OoGpr54^−/−^ mice (*lower panels*), gonadotropin priming resulted also in the appearance of newly formed corpora lutea, and cumulus–oocyte complexes in the oviduct. Previously cycling animals (**E**–**H**) showed the same features than control mice, with fresh corpora lutea (*CL1* in **E**, **G**), oocytes in the oviduct (*arrows* in **B**) and corpora lutea of previous cycles (*CL2*, *CL3* in **E**, **H**). In non-cycling animals (**H**, **I**), the occurrence of POI was evidenced by the presence of only one generation of newly-formed corpora lutea (*CL1* in **I**, **K**), lack of regressing corpora lutea of previous cycles, and larger interstitial areas (**I**). Cumulus-oocyte complexes were present in the oviduct (*arrow* in **J**). Occasional unruptured, partially luteinized, cystic follicles (*LF*) with central hemorrhage (**L**) and containing the oocyte (*arrow* in **M**) were observed. In **N**, quantification of the number of newly formed CL per ovary (matching the number of oocytes in the oviducts) is presented for all groups; n = 7/group (control and OoGpr54^−/−^ mice); the latter with the following distribution: three and four for cycling and non-cycling OoGpr54^−/−^ mice, respectively. H&E, hematoxylin and eosin; POI, premature ovulatory insufficiency.

We explored whether the state of anovulation caused by oocyte-specific ablation of *Gpr54* could be reversed by conventional gonadotropin priming at an incipient stage of the instauration of the POI-like phenotype, i.e. at 4 months of age. Seven OoGpr54^−/−^ mice were subjected to gonadotropin priming, consisting of a dose of PMSG followed by an ovulatory dose of hCG after 47 h, and sampling after 24 h. Histological analyses of the ovaries were used to discriminate between OoGpr54^−/−^ mice that were anovulatory or not before priming. Four OoGpr54^−/−^ mice (57%) did not show morphological signs of previous generations of CL, together with the presence of larger amounts of interstitial tissue, denoting a POI-like state. In contrast, the other three OoGpr54^−/−^ females (43%) did have histological evidence of previous generations of CL, and ovarian morphological features grossly identical to control mice. Regardless of their prevailing ovulatory state, all OoGpr54^−/−^ mice ovulated in response to gonadotropin priming, as denoted by the appearance of fresh (newly formed) CL in ovarian sections and by the presence of cumulus–oocyte complexes in the oviduct. In some non-cycling animals, occasional cystic, partially luteinized follicles, showing hemorrhagic signs in the central cavity, and containing the oocyte entrapped, were found. Quantification of the total number of fresh CL per ovary after gonadotropin priming revealed similar ovulatory responses in control and cycling OoGpr54^−/−^ mice; a modest trend for lower numbers of CL in response to priming was observed in non-cycling OoGpr54^−/−^ mice, albeit this was not significant (Fig. 5). Of note, serum AMH levels after completion of the gonadotropin priming protocol were significantly lower in OoGpr54^−/−^ mice, despite effective induction of ovulation (Supplementary Fig. S5).

## Discussion

POI is a relatively frequent condition, affecting ∼1% of women in reproductive age, defined by the loss of ovulatory capacity before the age of 40 years (Shelling, 2010; Huhtaniemi *et al.*, 2018). Clinical attention to POI has increased substantially in recent years, due to the switch in human reproductive habits to later age at first pregnancy. Clinical presentations of POI are diverse, including primary amenorrhea, representing only 10% of cases (Rebar and Connolly, 1990), secondary amenorrhea or oligomenorrhea for >4 months (Nelson, 2009). Iatrogenic and toxic forms of POI have been described. In addition, up to 30% cases of POI seem be autoimmune (Silva *et al.*, 2014), while the prevalence of familiar forms of POI ranges between 4% and 31% of cases. The advent of next generation sequencing has allowed the identification of an ever growing number of genes that can be causative of POI, via multiple pathways (Huhtaniemi *et al.*, 2018). Yet, >70% of the diagnosed cases of POI remain of unknown origin, referred as idiopathic (Shelling, 2010). Cases of POI concentrate mostly between 30 and 40 years of age, so that incidence of POI <30 years is only 0.1% of all women (Beck-Peccoz and Persani, 2006). Early diagnosis of POI has been advocated as key for an effective, personalized management of the condition (Huhtaniemi *et al.*, 2018), which can be reversible in a fraction of cases during early stages after instauration, but very seldom in situations of long-term amenorrhea.

**Figure 6.**
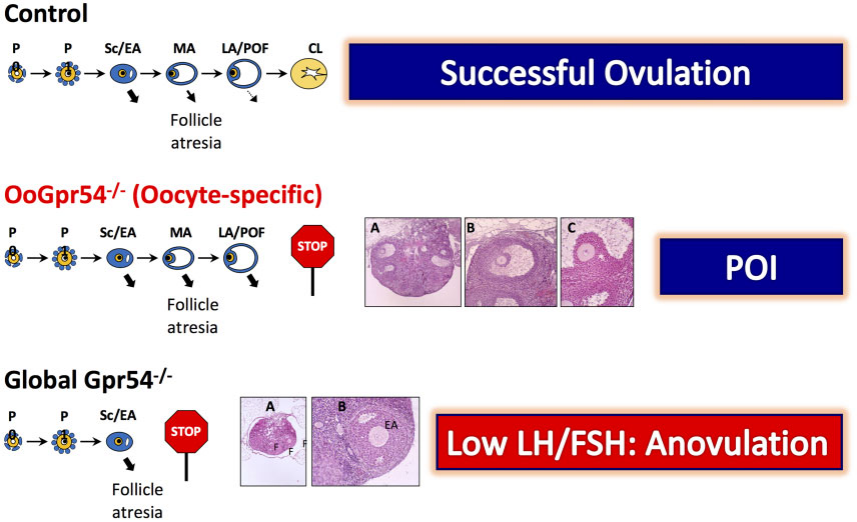
**Progressive premature ovarian insufficiency in OoGpr54^−/−^ mice.** Schematic representation of the proposed mechanism of premature ovulatory insufficiency (POI) caused by selective ablation of Gpr54 in oocytes. In comparison with control mice, in which follicular maturation leads to cyclic ovulation, despite some degree of follicular atresia, specially at the transition from Secondary/Early Antral (Sc/EA) to Mid- and Large-Antral follicles, in OoGpr54^−/−^ mice, increased follicular atresia at late stages of folliculogenesis results in progressive failure of ovulation at a relatively early age (4–6 months). In clear contrast, our previous data have documented that in global Gpr54^−/−^ mice failure of ovulation is constitutive (actually, animals do not enter puberty) and is mainly due to massive follicular atresia at the Sc/EA stage, caused by low LH and FSH levels. In both oocyte-specific and global Gpr54^−/−^ mice, ovulation can be rescued by appropriate gonadotropin priming.

We present here physiological evidence of a relevant role of kisspeptin signaling in the oocyte in maintaining proper ovulatory dynamics during the reproductive lifespan, whose ablation results in a progressive form of ovulatory failure, reminiscent of some key features of POI (for a graphical summary, see Fig. 6). As yet fragmentary data had suggested that local kisspeptin signaling in the ovary might modulate different ovarian functions, including granulosa cell steroidogenesis and hormone response, follicular dynamics, ovulation and reproductive aging (Dorfman *et al.*, 2014; Gaytan *et al.*, 2014; Owens *et al.*, 2018). However, available evidence was mostly indirect and has not permitted proper dissection of direct versus gonadotropin-mediated actions of kisspeptins. Specifically, actions of kisspeptins in the oocyte have been suggested by the demonstration of oocyte expression of *Gpr54* in various species (Saadeldin *et al.*, 2012; Dorfman *et al.*, 2014; Cielesh *et al.*, 2017). Furthermore, interaction of kisspeptins with neurotrophin signaling in the oocyte, mediated via the receptor, NTRK2, has been proposed to be essential for maintenance of follicular assembly and oocyte survival (Dorfman *et al.*, 2014). Yet, no conclusive evidence for a direct, discernible role of kisspeptin actions in the oocyte *in vivo* had been presented to date. Our current data disclose that, while direct kisspeptin actions are grossly dispensable for puberty onset and initial ovulation, preserved oocyte-specific actions of kisspeptins are mandatory to maintain full ovulatory capacity during the reproductive lifespan and to prevent excessive atresia of large antral follicles, thereby avoiding premature ovarian failure. These data support a tenable crosstalk between locally-produced kisspeptins and the oocyte to fine tune key aspects of the ovulatory process. Although the precise cellular source of such locally born kisspeptins is yet to be defined, our data point out that perturbation of such a local crosstalk would compromise follicle dynamics, favoring follicle atresia and disruption of ovulation. Among other sources, there are reports for expression of kisspeptins in granulosa and/or theca cells of the follicular wall in various species (Castellano *et al.*, 2006; Gaytan *et al.*, 2009; Hu *et al.*, 2017), and expression of *Kiss1* in the ovary has been shown to increase during pubertal maturation and the pre-ovulatory period in rats (Castellano *et al.*, 2006). Based on our present data, these locally born kisspeptins are tenable to drive a signal to the oocyte that contributes to prevention of follicular atresia and completion of ovulation during the reproductive lifespan. Indeed, evidence for the capacity of kisspeptins to promote oocyte maturation *in vitro* has been presented very recently, thereby supporting the physiological relevance of the signaling pathway interrogated in our work *in vivo* (Chakravarthi *et al.*, 2021). Apart from their physiological dimension, our data consolidate the possibility of direct oocyte actions of systemically-delivered kisspeptins, which might be relevant form a clinical standpoint, since kisspeptins have been recently advocated as potential egg maturation and ovulatory agents in assisted-reproductive technologies (Jayasena *et al.*, 2014; Abbara *et al.*, 2017).

While our OoGpr54^−/−^ mouse line unambiguously displayed early anovulation and decrease of circulating estrogen levels, both features compatible with POI, we did not detect changes in circulating LH or FSH levels in single determinations at any age-point analyzed. In one hand, this feature suggests that the central gonadotropin drive is preserved in our null model, and hence that the ovulatory failure would not be caused by defective gonadotropin stimulation but rather by a primary alteration at the ovarian level. On the other hand, it discloses a hormonal disconnection between gonadotropin and sex steroid levels, without detectable elevation of serum FSH, which is characteristic of clinical forms of POI. The underlying reasons are yet to be solved, but one tenable explanation is that the histological substrate of the ovulatory defect in our model, which is dominated by atresia of large antral follicles, without rapid exhaustion of the follicular pool, might prevent the overt elevation of gonadotropin levels, in contrast to more common forms of POI, in which accelerated elimination of the follicular reserve is bound to increase FSH concentrations. In addition, the trend for decreased AMH levels in OoGpr54^−/−^ mice at 10 months of age might also contribute to avoid the elevation of gonadotropins at this late age-point, since AMH has been recently shown to play an stimulatory role in the central control of gonadotropin secretion (Cimino *et al.*, 2016). Future studies, e.g. addressing pituitary function in our model, might help to further define the basis for this phenomenon. On the other hand, the trend for lower AMH levels in 10-month-old OoGpr54^−/−^ mice, as well as the significant reduction of circulating AMH after gonadotropin priming in null animals, could be indicative that some subtle failure at the level of small follicles, as major source of AMH, might occur in our model, but this would become only evident after ovarian challenge (e.g. with gonadotropins) or incipient aging. The pathophysiological relevance of such perturbation of circulating AMH is yet to be defined.

The fact that the penetrance of the POI-like phenotype in OoGpr54^−/−^ mice is incomplete and progresses with age might be due to different factors, including variable *Cre* expression and *Gpr54* ablation across the follicular pool and between different individuals. In any event, RT-qPCR analyses in isolated oocytes from 4-month-old OoGpr54^−/−^ mice documented >75% reduction in *Gpr54* mRNA levels, while direct RNA sequencing revealed close to undetectable levels of *Gpr54* transcripts in all but one OoGpr54^−/−^ mice at 6 months. Yet, it must be noted that ovarian sources of Gpr54 expression other than the oocyte have also been described (Hu *et al.*, 2017). Altogether, our data claim for an effective ablation of *Gpr54* in our model, and indicate a role of oocyte-specific actions of kisspeptin in the fine-tuning of late stages of follicular maturation/survival and ovulation, in cooperation with, and possibly subordinated to, other regulatory factors, including gonadotropins. Anyhow, the fact that removal of *Gpr54* from oocytes caused ovulatory failure attests for the physiological relevance of such direct oocyte actions in the long-term maintenance of fertility.

Comparison of our present findings with other mouse models of premature ovarian failure due to conditional ablation of several structural or regulatory factors in oocytes reveals distinct features of our OoGpr54^−/−^ line, in relation with the primary defects and time-course of this form of POI. Thus, elimination of intracellular signaling factors, such as PTEN (Phosphatase and Tensin Homolog delted on Chromosome 10), PDK1 (Phosphoinositide-dependent Kinase 1) and mTOR (mammalian Target of Rapamycin), which are essential to maintain the quiescence state of primordial follicles, resulted in premature activation of primordial follicle growth, leading to complete exhaustion of the ovarian follicular reserve and infertility before 3 months of age (Reddy *et al.*, 2008, 2009; Adhikari *et al.*, 2010). Likewise, oocyte-specific ablation of *Ntrk2* resulted in complete POI by 14 weeks, with total disintegration of follicular structure and oocyte loss (Dorfman *et al.*, 2014), while genetic elimination of the enzymatic machinery for the synthesis of complex O- and N-glycans in the oocyte resulted in subfertility already by 6 weeks, and complete ovarian failure and infertility between 9 and 11 weeks of age (Williams and Stanley, 2011; Grasa *et al.*, 2016). Yet, in the latter model, the primordial follicle pool remained unaltered, but there were signs of altered follicular development, including the appearance of morphologically abnormal large antral follicles that failed to ovulate (Williams and Stanley, 2011; Grasa *et al.*, 2016). In our model, progressive instauration of ovulatory failure resulted in a complete anovulatory state at much later stages, namely, between 6 and 10 months of age, which was defined by lack of CL and increased rate of antral follicular atresia. However, the total number of resting or early growing follicles, or even large follicles, was not overtly altered in OoGpr54^−/−^ mice. While common forms of POI can involve a rapid decline of small and antral follicles, resulting in follicular exhaustion and early menopause, our histological findings are compatible with the clinical presentation of >50% cases of POI in humans, named follicular POI, where variable populations of follicles, including antral follicles, are present (Nelson, 2009), therefore suggesting the occurrence of follicular dysfunction, rather than total depletion (Grasa *et al.*, 2016). On the other hand, the temporal course of ovulatory failure in our model more closely resembles that of most of the human cases of POI, which occur beyond 30 years of age (Beck-Peccoz and Persani, 2006), and further emphasizes the value of our OoGpr54^−/−^ mouse to study specific aspects of ovulatory dynamics, and its alteration in some forms of the human disease.

We applied direct RNA sequencing to whole ovarian tissue to identify novel molecular mechanisms and markers of the POI-like condition of our model. Admittedly, single-cell RNAseq analyses might have allowed more robust identification of oocyte-specific deregulated gene pathways, but this approach would have precluded capturing markers of altered communication between the oocyte and surrounding somatic cells, which seems to be determinant for the ovulatory failure in OOGpr54^−/−^ mice. On the other hand, transcriptomic analyses in isolated antral follicles might have better assessed changes in the intra-follicular crosstalk in our mouse line but would have missed changes occurring at ovarian somatic cells outside follicles. These features, together with technical limitations to obtain sufficient, high-quality oocyte RNA without gonadotropin priming or to ensure accurate dissection of homogeneous antral follicle populations, prompted us to opt for whole ovary analyses that, despite being partially influenced by the anovulatory phenotype, do permit an integral assessment of alterations caused by oocyte ablation of kisspeptin signaling on the different ovarian compartments. Consistent with the split phenotype, with anovulatory and ovulatory KO mice up to the age of 6 months, large-scale transcriptomic analyses allowed identification of both phenotype- and genotype-driven differences in the RNA expression profiles of OoGpr54^−/−^ ovaries. Clustering analyses revealed that the RNA signature of ovaries lacking *Gpr54* in the oocytes is dominated by the phenotype; i.e. ovulatory KO mice are closer to controls than to anovulatory OoGpr54^−/−^ mice. This is possibly due to the impact of the presence or absence of CL transcriptome on the relative abundance of the remaining ovarian transcripts, as nicely illustrated by the expression profiles of gene markers of luteinization, as a hallmark process of ovulation, which were dramatically suppressed only in KO animals with an anovulatory phenotype. Notwithstanding, the expression of a subset of 21 genes displayed significant upregulation or downregulation in the ovaries of OoGpr54^−/−^ mice, regardless of the ovulatory state, suggesting that these changes are not secondary, but seemingly precede and might contribute to the POI-like phenotype. Among those, our analyses identified a significant suppression of the ovarian expression of *Adcyap1*, the gene encoding pituitary adenylate cyclase activating polypeptide (PACAP). PACAP is produced in mouse granulosa and cumulus cells, where it operates as local regulator of ovarian gonadotropin actions. Upon LH surge, PACAP stimulates cAMP formation and the production of progesterone and prostaglandin E_2_ in granulosa cells (Gras *et al.*, 1996; Lee *et al.*, 1999), and contributes to the induction of the follicular rupture (Stouffer *et al.*, 2007). Notably, kisspeptin is known to upregulate the expression on *Adcyap1* in primary cultures of rat placental cells (Oride *et al.*, 2016). Altogether, it is tenable that suppressed PACAP expression following ablation of kisspeptin signaling in oocytes might contribute to the ovulatory failure in our mouse model.

Other transcripts significantly downregulated in OoGpr54^−/−^ ovaries were *Abcb1b* and *Wnt-10b*, whose causative relation with POI had not previously been suggested. The AbcB1 family of multidrug resistance transporters are ATP-binding cassette transporters that efflux amphipathic cations from cells and protect tissues from xenobiotics; *Abcb1b* being the most abundantly expressed in mouse ovary (Cui *et al.*, 2009), whose levels cycle through estrus, in relation to estrogen surges (Brayboy *et al.*, 2017). Mice deficient for *Abcb1a*, *1b* and *Abcb2* have been reported to have fewer CL in their ovaries, accelerated follicular atresia, and elongated estrus phase of the cycle, but no difference in circulating hormone levels (Brayboy *et al.*, 2017); features that are partially reminiscent of the OoGpr54^−/−^ phenotype. On the other hand, members of the canonical WNT signaling pathway have previously been shown to play a major role in mammalian gonadal development, follicular maturation and ovarian steroidogenesis (Hernandez Gifford, 2015). Yet, limited evidence for the putative ovarian role of Wnt-10 has been presented to date, apart from the observed expression of Wnt-10a and-10b in the mouse ovary during the postnatal period and in the full-grown oocyte (Harwood *et al.*, 2008). The observed downregulation of *Wnt-10b* in OoGpr54^−/−^ ovaries might indicate a role of this member of the WNT family in POI, which is yet to be substantiated.

In contrast, OoGpr54^−/−^ ovaries displayed significant upregulation of a set of genes, which includes chloride channel accessory 2 (*Clca2*), the vitamin A-related genes, *1300002K09Rik* and *Aldh1a1* and the matrix-metalloprotease family member, *Mmp-12*. The potential connection of these factors and POI has not been previously defined, but their reported physiological roles are suggestive of a possible link. For instance, *1300002K09Rik* encodes the retinol-binding protein, RBPR2 (Alapatt *et al.*, 2013), whereas *Aldh1a1* codes for an aldehyde dehydrogenase (Conaway *et al.*, 2013), responsible for retinal oxidation into retinoic acid (RA). *Aldh1a1* is expressed in the ovary and its production can be increased by gonadotropin priming (Kawai *et al.*, 2012). Hence, the consistent upregulation of *1300002K09Rik* and *Aldh1a1* might perturb RA homeostasis in OoGpr54^−/−^ ovaries, leading to defective oocyte development and ovulation, in line with previous literature (Kawai *et al.*, 2016). Alike, substantial upregulation of *Mmp-12* was detected in OoGpr54^−/−^ ovaries; MMP-12 is a macrophage-produced matrix metalloprotease (MMP), with potent elastase activity, which has been involved in tissue remodeling, but whose ovarian role remains unknown. Our results suggest it may contribute to follicular atresia, rather than ovulation or luteal formation and regression, where other MMPs dominate (Curry and Osteen, 2001).

Notably, phenotype-driven differences in the RNA expression profiles of OoGpr54^−/−^ ovaries were notable and included 161 differentially expressed genes, which were either upregulated (n = 47) or downregulated (n = 144) in the POI-like condition. In addition to evident CL-associated transcripts (see Fig. 2c), KEGG analyses of this gene dataset allowed identification of biological pathways putatively activated or repressed in ovaries undergoing anovulatory failure in our model. Among the upregulated pathways, changes in ovarian redox systems (as denoted by enhancement of glutathione and peroxisome related pathways) and AGE-RAGE signaling are of particular interest, as ovarian aging has been shown to induce an enhancement of oxidative stress at the follicular microenvironment (Pertynska-Marczewska and Diamanti-Kandarakis, 2017), that may contribute to ovulatory dysfunction. Interestingly, accumulation of AGE in ovarian follicles has been proposed to trigger early ovarian aging (Pertynska-Marczewska and Diamanti-Kandarakis, 2017; Gill *et al.*, 2019); our data being the first evidence suggestive of a putative link between defective kisspeptin signaling in the oocyte and activation of AGE-RAGE signaling in the context of POI. In addition, the presumptive activation of PI3K/Akt signaling in the ovaries of OoGpr54^−/−^ mice undergoing ovulatory failure is worth of attention, given the connection of this signaling pathway with ovarian PTEN (Makker *et al.*, 2014), whose deregulation leads also to POI (Reddy *et al.*, 2008), albeit with different phenotypic manifestation.

On the other hand, ovarian downregulation of essential cellular homeostasis mechanisms (e.g. lysosomal and phagosome pathways), PPAR signaling and cytochrome P450-related xenobiotic metabolism was inferred on the basis of KEGG analyses in OoGpr54^−/−^ mice displaying premature anovulation. While a causative link between these changes and POI is not conclusively demonstrated by our data, it is worth noting that PPAR signaling in the ovary has been shown to influence granulosa-oocyte crosstalk and is involved in ovulation (Komar, 2005; Minge *et al.*, 2008; Vitti *et al.*, 2016). Hence, the suppression of this pathway, caused by selective elimination of kisspeptin signaling in the oocyte, may contribute to the POI-like phenotype. Similarly, since exposure to xenobiotics has been linked to ovotoxicity and early ovarian failure (Hoyer and Keating, 2014), it is tenable that the suppression of their metabolism machinery in the ovary may favor premature ovarian failure in our model. In the same vein, the dramatic suppression of the lysosome pathway in the ovaries of anovulatory OoGpr54^−/−^ mice might resemble conditions of lysosomal dysfunction, some of which are associated to accelerated ovulatory failure in mice (Wang *et al.*, 2019). The putative mechanisms connecting the perturbation of kisspeptin signaling in the oocyte and the above alterations warrant future investigation.

Finally, our data also demonstrate that the ovulatory failure of OoGpr54^−/−^ mice can be rescued by appropriate gonadotropin priming, at least at early stages of development of the phenotype. While the prevalent consensus is that gonadotropin priming has little benefit in common, consolidated forms of POI, some clinical studies have indeed demonstrated that a fraction of women with POI can actually respond to gonadotropins in terms of ovulation (Badawy *et al.*, 2007; Tartagni *et al.*, 2007), albeit responses in those studies were variable and might be determined by the prevailing histological substrate. In any event, this feature of our model is reminiscent of some clinical presentations of POI, in which recurrence of ovulation and pregnancy can occur, even spontaneously, short after instauration of amenorrhea. The mechanism for such rescue is yet to be disclosed, but it is tenable to hypothesize that a strong gonadotropin drive may overcome the subtle defect imposed by the interruption of local kisspeptin-oocyte crosstalk, at least with the pharmacological protocol applied at this early stage. Of note, in contrast to other forms of POI, our mouse model might be specially prone to ovulatory rescue, as indirectly suggested by the fact that global Gpr54 null mice could be forced to ovulate, if intensively primed with gonadotropins, although ovulatory responses to gonadotropins were quantitatively sub-normal in whole-body Gpr54 null mice (Gaytan *et al.*, 2014).

In sum, we provide herein evidence for a physiological role of kisspeptin signaling specifically in the oocyte in the control of ovulation and follicle survival during the reproductive lifespan, whose deregulation may lead to a progressive form of premature ovarian failure. Admittedly, while our model of conditional ablation of Gpr54 in the oocyte unequivocally displays premature ovulatory failure, it does not phenocopy all clinical manifestations of prevalent forms of POI in humans, as it does not result in overt depletion of the ovarian reserve and is prone to ovulatory rescue following appropriate gonadotropin priming. However, since the heterogeneity and clinical course of this condition are not fully captured either by many of the available genetic models of POI, we believe our OoGpr54^−/−^ mouse may represent a useful complementary tool for investigation of the molecular basis and eventual novel therapies of some forms of this prevalent disease.

## Data availability

The data underlying this article will be shared on reasonable request to the corresponding author.

## Supplementary Material

deab287_Supplementary_Figure_S1

deab287_Supplementary_Figure_S2

deab287_Supplementary_Figure_S3

deab287_Supplementary_Figure_S4

deab287_Supplementary_Figure_S5

deab287_Supplementary_Figure_S6

deab287_Supplementary_Figure_S7

deab287_Supplementary_Figure_S8

deab287_Supplementary_Table_S1

deab287_Supplementary_Table_S2
