# Supplementary material for: Selective loss of kisspeptin signaling in oocytes causes progressive premature ovulatory failure
Source: Hum Reprod. 2022 Jan 17;37(4):806–21. doi: 10.1093/humrep/deab287 (PMC8971646; doi:10.1093/humrep/deab287)
Supplement: deab287_Supplementary_Figure_S1 [file deab287_supplementary_figure_s1.pdf]

Human Reproduction, pp. 1–1, 2022
https://doi.org/10.1093/humrep/deab287

human reproduction

SUPPLEMENTARY DATA

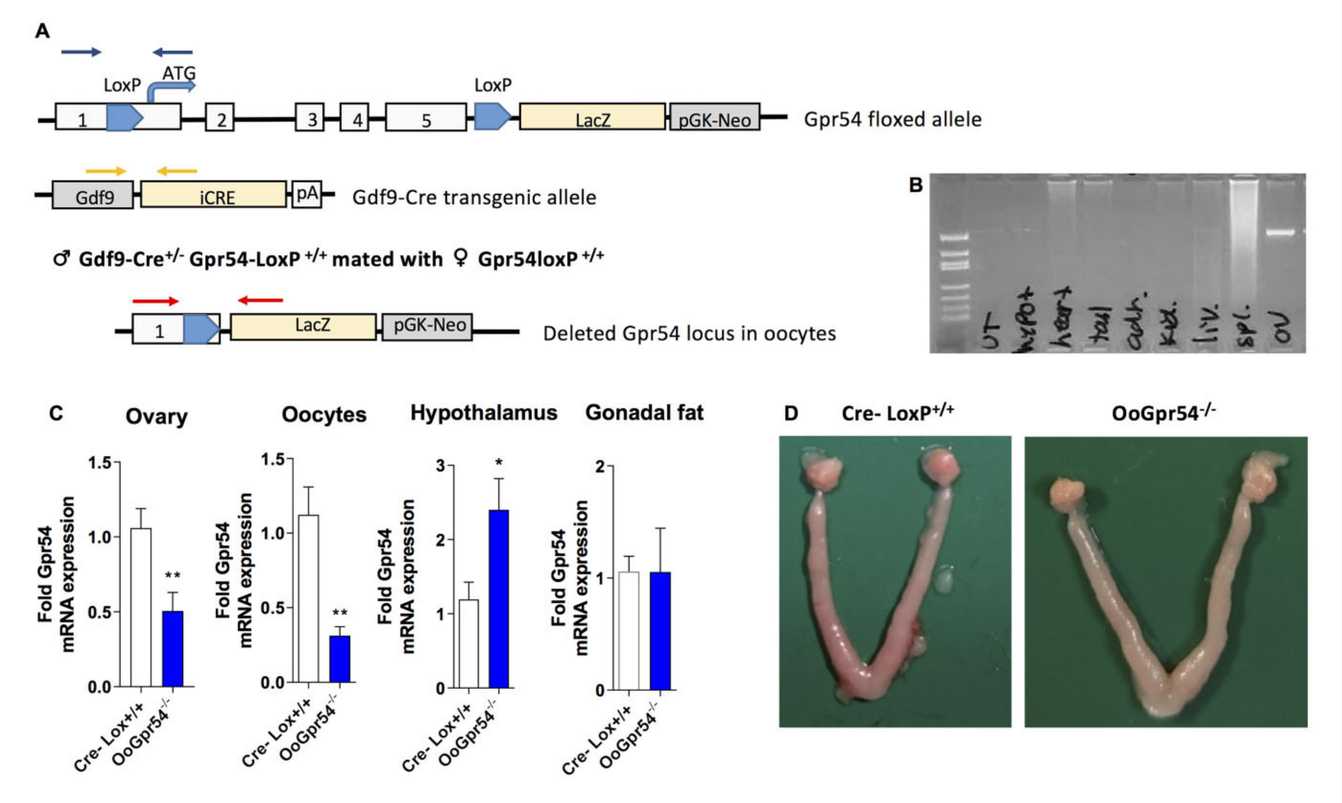

Supplementary Figure S1. Generation of OoGpr54$^{-/-}$ mice. In panel (**A**), the strategy for generation of oocyte-specific deletion of *Gpr54*, by heterozygous crossing of a Gdf9-iCre/Gpr54-LoxP double mutant line. Arrows indicate the location of the genotyping primers to detect the LoxP sites (blue), the iCre transgene (yellow) and the DNA recombination (red). In panel (**B**), PCR results to show a Cre-driven DNA recombination only in the ovary (OV), and not in the uterus (UT), hypothalamus (HY), heart (HE), tail (TA), adrenals (AD), kidney (KI), liver(LI) or spleen (SP). In panel (**C**), expression levels of *Gpr54* mRNA in the ovary, isolated oocytes, the hypothalamus and gonadal fat; values are presented as fold-change of OoGpr54$^{-/-}$ levels versus controls (Cre^{-}LoxP$^{+/+}$). In panel (**D**), gross morphological appearance of the uterus and ovaries in 4-month-old control and knock-out animals. Values are means ± SEM, n = 5–13/group. *P*-values with Student's *t*-test: $*P < 0.05$ and $**P < 0.01$.
