# Supplementary material for: Selective loss of kisspeptin signaling in oocytes causes progressive premature ovulatory failure
Source: Hum Reprod. 2022 Jan 17;37(4):806–21. doi: 10.1093/humrep/deab287 (PMC8971646; doi:10.1093/humrep/deab287)
Supplement: deab287_Supplementary_Figure_S2 [file deab287_supplementary_figure_s2.pdf]

Human Reproduction, pp. 1–1, 2022
https://doi.org/10.1093/humrep/deab287

human reproduction

SUPPLEMENTARY DATA

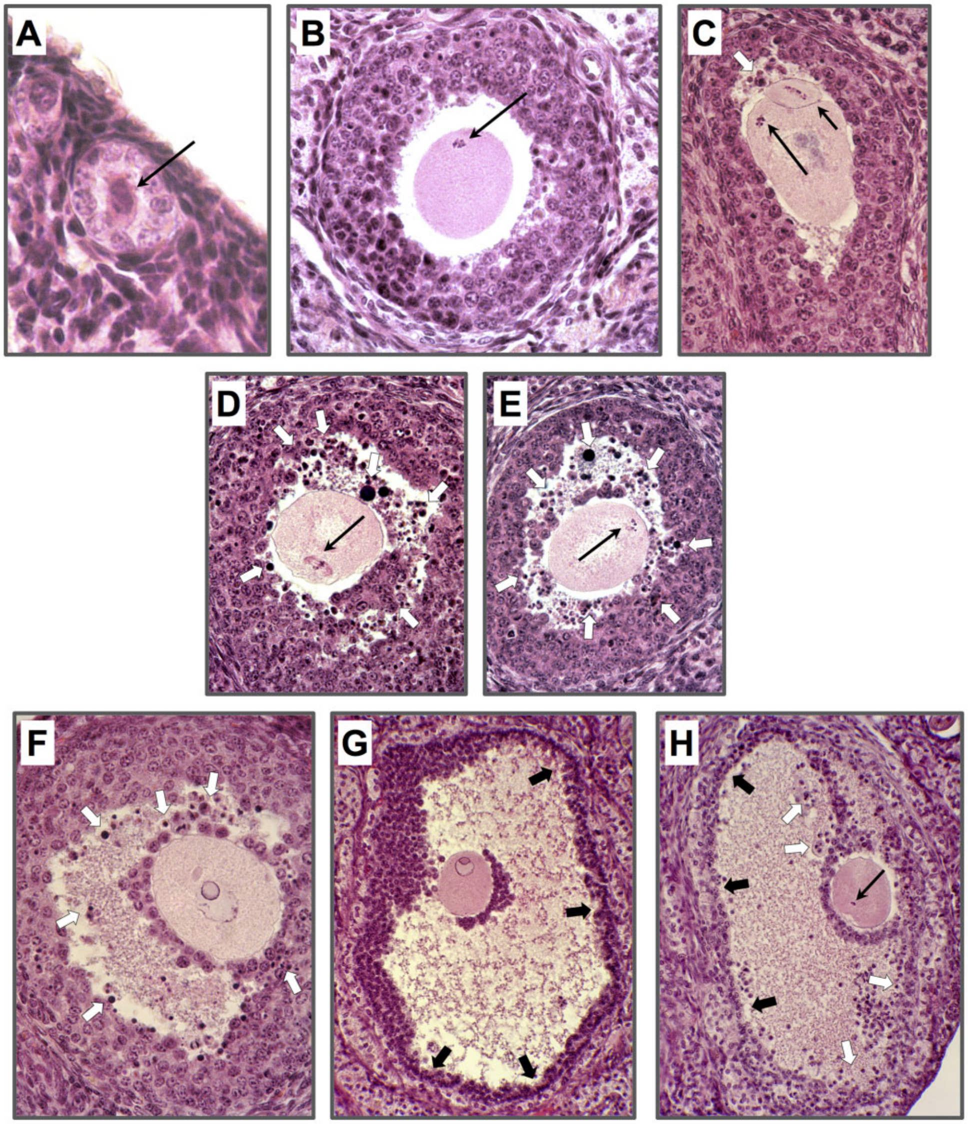

Supplementary Figure S2. Features of follicular atresia in OoGpr54$^{-/-}$ mice. Follicle atresia in OoGpr54$^{-/-}$ animals displayed patterns similar to those of control rodents. Small (preantral) follicles showed oocyte alterations, such as cytoplasmic shrinkage (**A**), meiotic spindle (*large arrow* in **B**, **C**) or parthenogenetic fragmentation (*short arrow* in **C**), with minimal changes in the granulosa layer (*white arrow* in **C**). In transitional pre-antral/early antral follicles (**D**, **E**), both oocyte alterations (*large arrow*) and abundant pyknotic granulosa cells (*white arrows*) were present at early stages of atresia. In contrast, antral follicles at the early stages of atresia showed abundant pyknotic granulosa cells (*white arrows* in **F**), or thinning and deformation of the granulosa layer (*black arrows* in **G**), while the oocyte remained in the germinal vesicle stage, without evident morphological alterations. Morphological signs of oocyte degeneration, such as the presence of meiotic-like spindle (*large arrow* in **H**) and parthenogenetic fragmentation, were only observed at advanced stages of atresia, in follicles showing also abundant pyknotic granulosa cells (*white arrows*) and general thinning and disorganization of the granulosa cell layer (*black arrows*).
