# Supplementary material for: Selective loss of kisspeptin signaling in oocytes causes progressive premature ovulatory failure
Source: Hum Reprod. 2022 Jan 17;37(4):806–21. doi: 10.1093/humrep/deab287 (PMC8971646; doi:10.1093/humrep/deab287)
Supplement: deab287_Supplementary_Figure_S3 [file deab287_supplementary_figure_s3.pdf]

Human Reproduction, pp. 1–1, 2022
https://doi.org/10.1093/humrep/deab287

human reproduction

SUPPLEMENTARY DATA

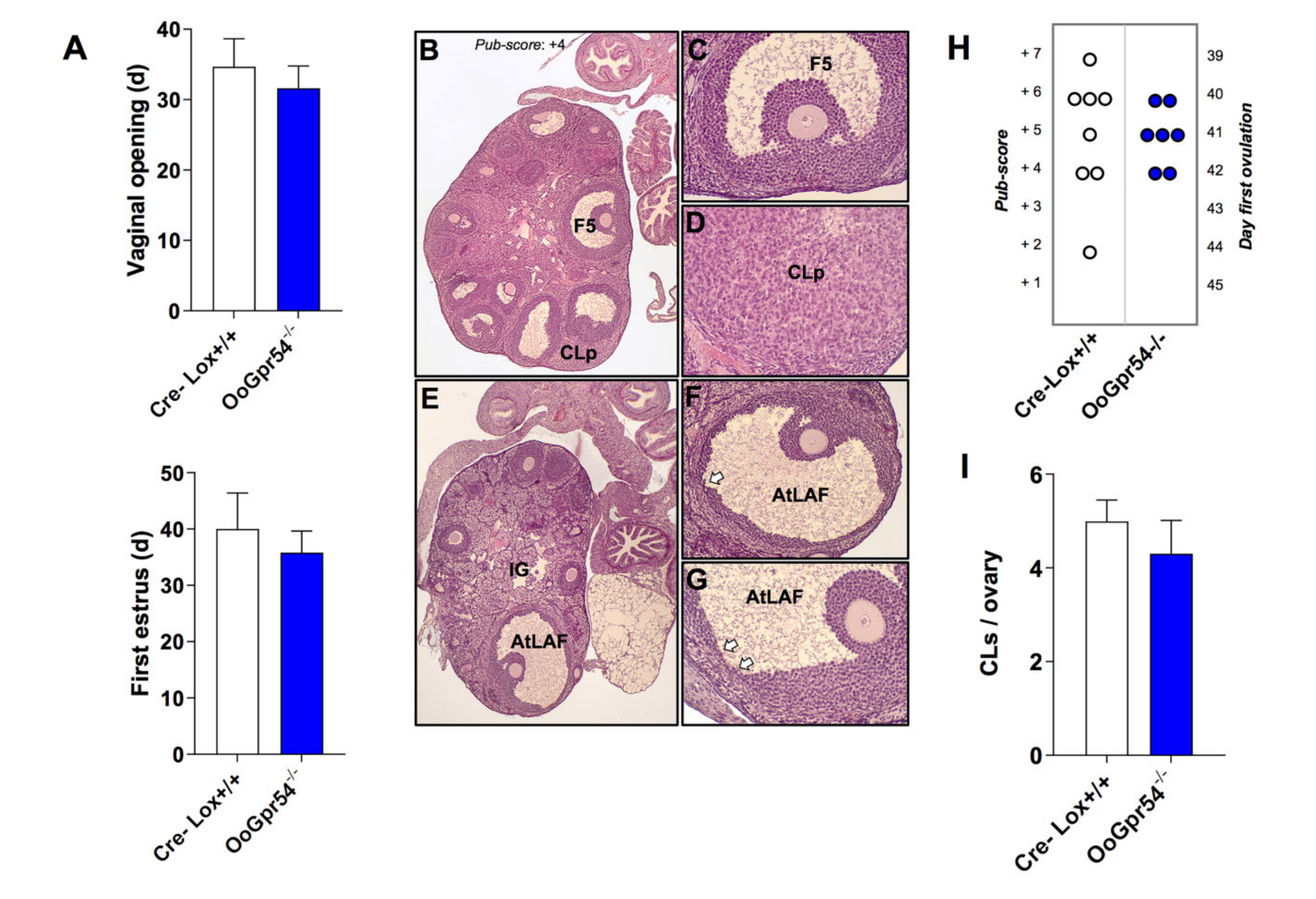

Supplementary Figure S3. Normal puberty onset in OoGpr54$^{-/-}$ mice. In panel (**A**), average post-natal day (PND) of vaginal opening (*top panel*), as phenotypic sign of the onset of puberty. The PND of the first estrus was also analyzed from vaginal smear samples (*bottom panel*). In panels (**B**–**D**), representative H&E staining of the ovary at PND45, assessed from 8 animals per genotype. In panels (**E**–**G**), H&E staining of the ovary at PND 45 of a knock-out female that has not yet ovulated. The ovary lacks corpora lutea and an atretic large follicle (AtLAF) is visible; white arrows denote thinning of the granulosa layer, as a sign of advanced follicle atresia. In panel (**H**), individual Pub-scores and day of the first ovulation values from each analyzed individual based on H&E staining of the ovaries at PND 45. In panel (**I**), average number of corpora lutea in ovaries at PND 45. Values are means ± SEM, n = 8/group. No significant differences were detected, except there was one OoGpr54$^{-/-}$ female that had no CL and, thus, was not included in the histogram. CL, corpus luteum; IG, interstitial gland; H&E, hematoxylin & eosin.
