# Supplementary material for: Selective loss of kisspeptin signaling in oocytes causes progressive premature ovulatory failure
Source: Hum Reprod. 2022 Jan 17;37(4):806–21. doi: 10.1093/humrep/deab287 (PMC8971646; doi:10.1093/humrep/deab287)
Supplement: deab287_Supplementary_Figure_S4 [file deab287_supplementary_figure_s4.pdf]

**Human Reproduction, pp. 1–1, 2022**
https://doi.org/10.1093/humrep/deab287

human reproduction

# SUPPLEMENTARY DATA

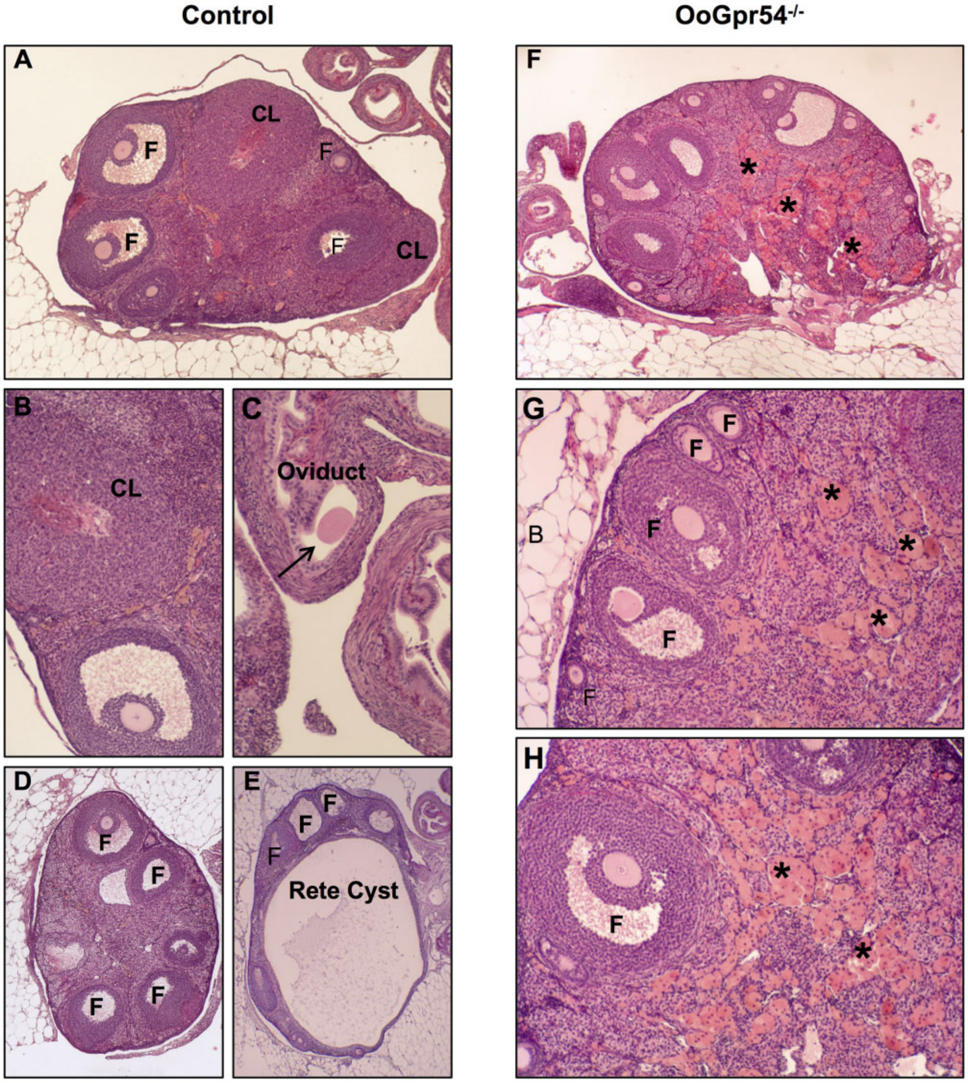


**Supplementary Figure S4. Ovarian histology in 10- to 11-month-old control and OoGpr54$^{-/-}$ mice.** In the left panels, representative photomicrographs of 11-month-old control, Cre-LoxP$^{+/+}$ mice are shown. While some individuals (3/9) showed fresh corpora lutea (CL), together with large, health antral follicles (F), with oocytes released in the oviduct, as direct signs of persistent ovulation (see panels **A**–**C**), other control mice of this age presented ovarian alterations consisting in irregular cycles, with absence of fresh CL, despite the presence of large antral follicles, together with other morphological abnormalities, such as cysts in the *rete ovarii*, suggesting incipient ovarian aging. Nonetheless, pigment-laden interstitial cells in control ovaries at this age were scarce, suggesting that ovarian alterations started only recently. In contrast, all pair-aged OoGpr54$^{-/-}$ mice (n = 8) displayed an anovulatory state, defined by absence of CL, and had signs of advanced ovarian aging, such as accumulation of residual pigments in the interstitial areas (i.e. pigment-laden macrophages; see asterisks in panels **F**–**H**). However, growing follicles were also present, indicating that ovarian insufficiency was not due to exhaustion of the ovarian reserve; see panels **G**–**H.**
