# Supplementary material for: Selective loss of kisspeptin signaling in oocytes causes progressive premature ovulatory failure
Source: Hum Reprod. 2022 Jan 17;37(4):806–21. doi: 10.1093/humrep/deab287 (PMC8971646; doi:10.1093/humrep/deab287)
Supplement: deab287_Supplementary_Figure_S5 [file deab287_supplementary_figure_s5.pdf]

Human Reproduction, pp. 1–1, 2022
https://doi.org/10.1093/humrep/deab287

human reproduction

SUPPLEMENTARY DATA

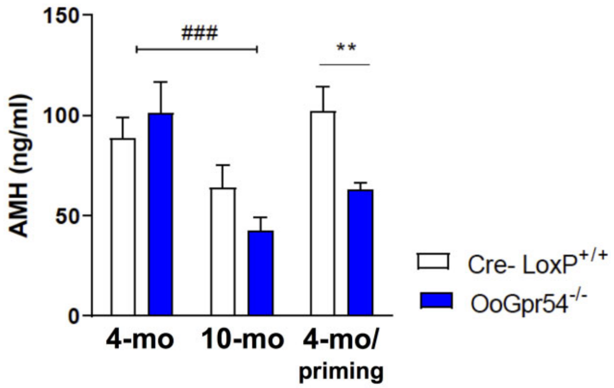

Supplementary Figure S5. Circulating AMH levels in OoGpr54$^{-/-}$ mice. Serum AMH levels in OoGrp54$^{-/-}$ and control (Cre^{-} LoxP$^{+/+}$) mice, at 4- and 10-month of age, and in 4-month-old OoGpr54$^{-/-}$ mice, and their corresponding controls, 24-h after completion of a protocol of gonadotropin (hCG) priming leading to ovulation, are presented. Values are means ± SEM, n = 5–11/group for 4- and 10-month-old mice, and n = 7/group for 4-month-old animals subjected to hCG priming. $^{###}P < 0.001$ versus the 4-month-old groups (two-way ANOVA); $^{**}P < 0.01$ versus corresponding control group (Student's *t*-test). AMH, anti-Mullerian hormone.
