# Supplementary material for: Selective loss of kisspeptin signaling in oocytes causes progressive premature ovulatory failure
Source: Hum Reprod. 2022 Jan 17;37(4):806–21. doi: 10.1093/humrep/deab287 (PMC8971646; doi:10.1093/humrep/deab287)
Supplement: deab287_Supplementary_Figure_S6 [file deab287_supplementary_figure_s6.pdf]

Human Reproduction, pp. 1–1, 2022
https://doi.org/10.1093/humrep/deab287

human reproduction

SUPPLEMENTARY DATA

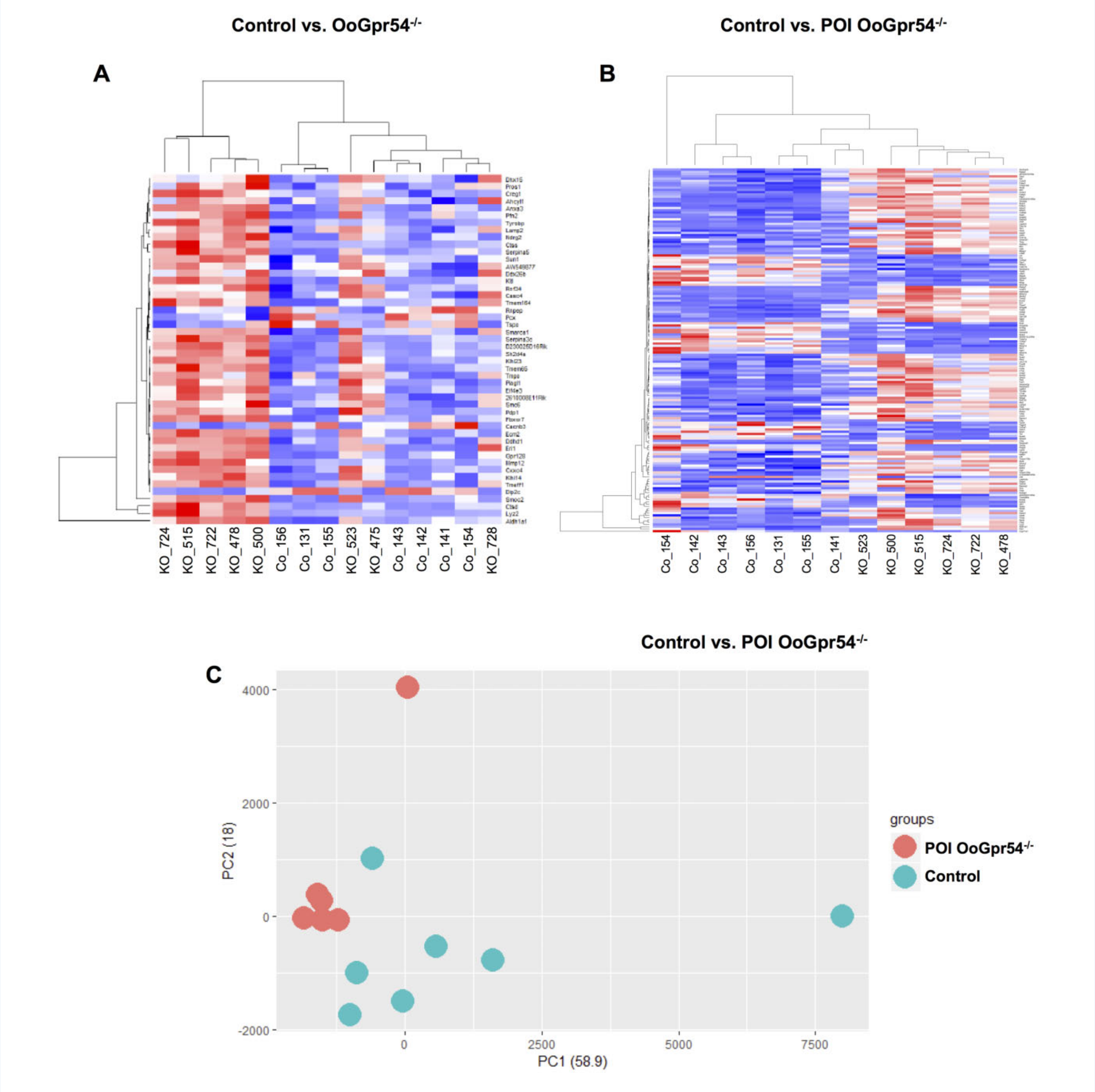

Supplementary Figure S6. Heatmap and principal component analyses of differentially expressed genes in OoGpr54$^{-/-}$ mice displaying premature ovulatory failure. In panel (**A**), a heatmap of differentially expressed genes between genotypes (control Cre-LoxP$^{+/+}$ versus OoGpr54$^{-/-}$ mice, irrespective of the POI phenotype) reveals an incomplete segregation, with the two KO individuals not showing anovulation (KO_475 and KO_728) clustering together with control animals. In contrast, a total of 161 differentially expressed genes were identified when comparing control and anovulatory OoGpr54$^{-/-}$ mice, including 47 upregulated and 114 downregulated in the POI condition. In panel (**B**), the heatmap showing the expression of these 161 genes is presented; this clustering analysis shows a perfect segregation between the control and the POI-like phenotype. In panel (**C**), the plot showing the first principal component analysis (PCA) for discrimination between control and POI OoGpr54$^{-/-}$ (KO) mice is displayed. POI, premature ovulatory insufficiency.
