# Supplementary material for: Selective loss of kisspeptin signaling in oocytes causes progressive premature ovulatory failure
Source: Hum Reprod. 2022 Jan 17;37(4):806–21. doi: 10.1093/humrep/deab287 (PMC8971646; doi:10.1093/humrep/deab287)
Supplement: deab287_Supplementary_Figure_S7 [file deab287_supplementary_figure_s7.pdf]

Human Reproduction, pp. 1–1, 2022
https://doi.org/10.1093/humrep/deab287

human reproduction

SUPPLEMENTARY DATA

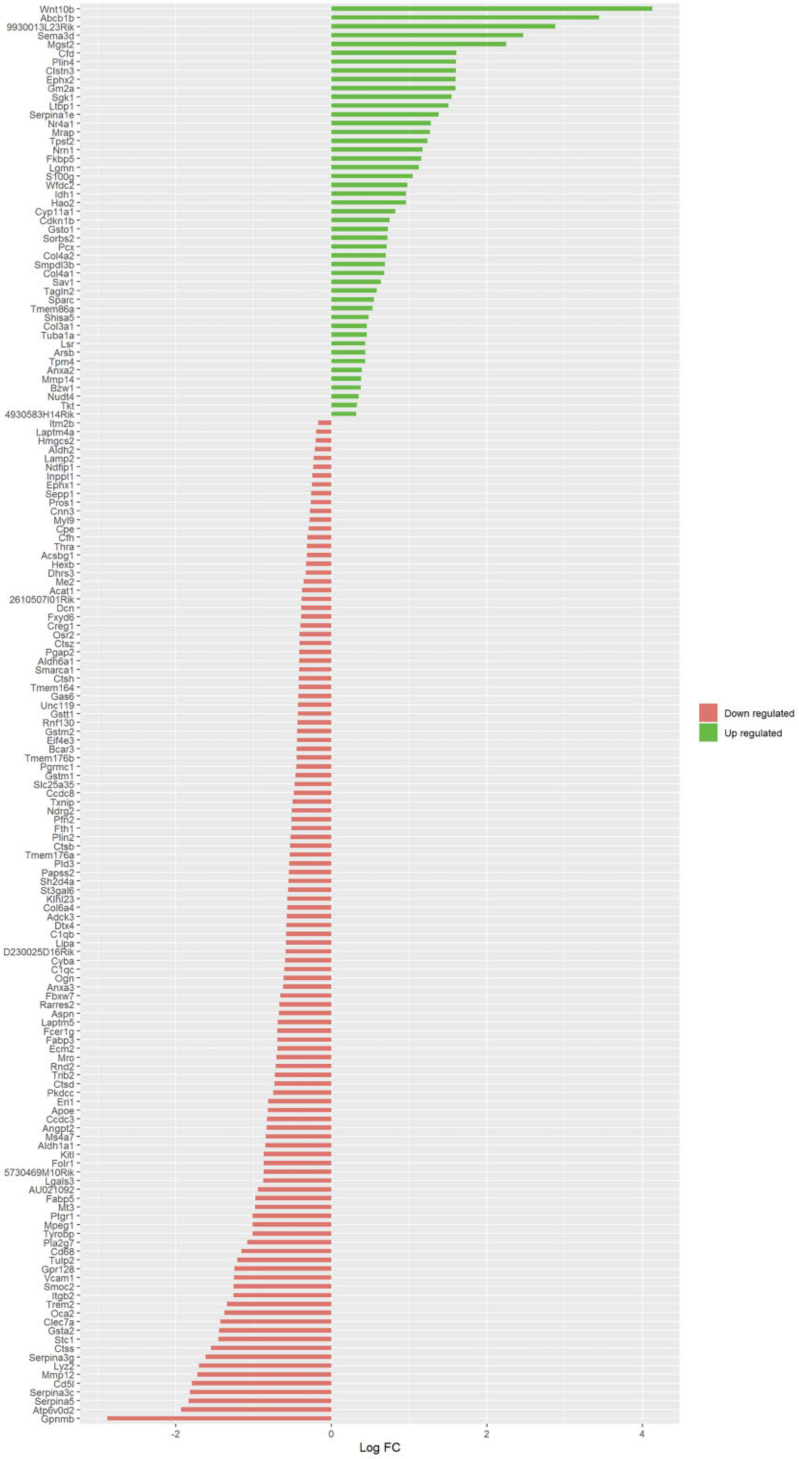

Supplementary Figure S7. Fold-changes of differentially upregulated and downregulated genes in the ovary of in OoGpr54$^{-/-}$ mice displaying premature ovulatory failure. Representation of log fold-changes of differentially expressed genes in the ovaries of anovulatory OoGpr54$^{-/-}$ mice. Upregulated genes (47) are displayed in green, while downregulated genes (114) are shown in red.
