# Supplementary material for: Selective loss of kisspeptin signaling in oocytes causes progressive premature ovulatory failure
Source: Hum Reprod. 2022 Jan 17;37(4):806–21. doi: 10.1093/humrep/deab287 (PMC8971646; doi:10.1093/humrep/deab287)
Supplement: deab287_Supplementary_Figure_S8 [file deab287_supplementary_figure_s8.pdf]

Human Reproduction, pp. 1–1, 2022
https://doi.org/10.1093/humrep/deab287

human reproduction

SUPPLEMENTARY DATA

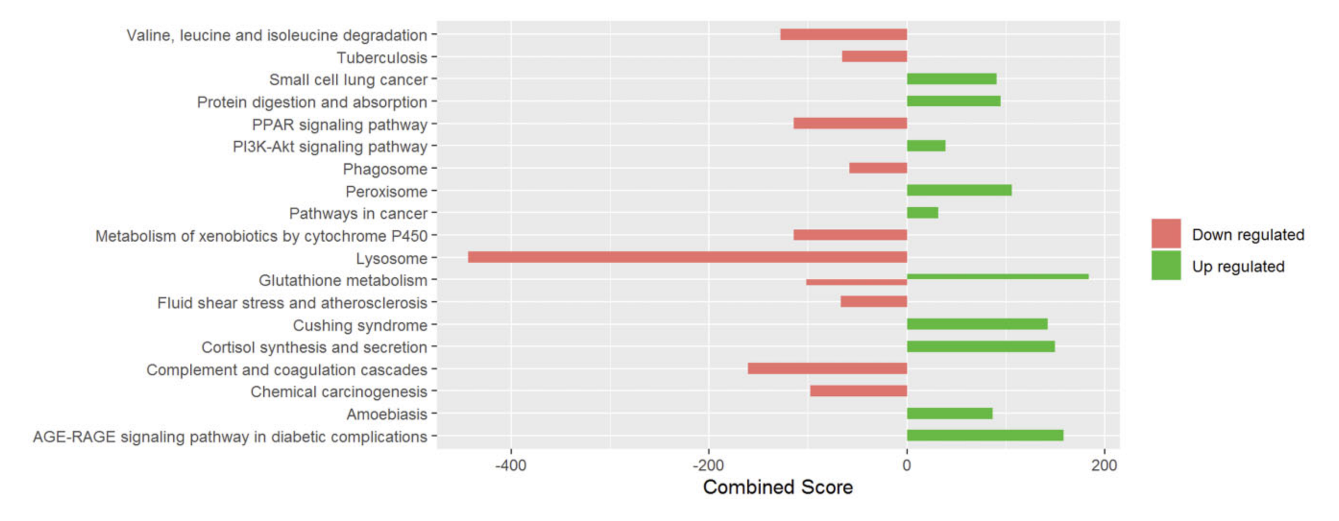

Supplementary Figure S8. KEGG pathway enrichment analyses in ovaries of OoGpr54$^{-/-}$ mice displaying premature ovulatory failure. KEGG pathway enrichment analysis representing the combined score calculated as $c = \log(p) * z$, where $c =$ the combined score, $p = p$-value and $z = z$-score for deviation from expected rank. KEGG, Kyoto Encyclopedia of Genes and Genomes.
