# Supplementary material for: Selective loss of kisspeptin signaling in oocytes causes progressive premature ovulatory failure
Source: Hum Reprod. 2022 Jan 17;37(4):806–21. doi: 10.1093/humrep/deab287 (PMC8971646; doi:10.1093/humrep/deab287)
Supplement: deab287_Supplementary_Table_S1 [file deab287_supplementary_table_s1.pdf]

Human Reproduction, pp. 1–1, 2022
https://doi.org/10.1093/humrep/deab287

human reproduction

SUPPLEMENTARY DATA

Supplementary Table SI List of primer pairs used for PCR and qPCR assays.

Target	Forward	Reverse	Amplicon size (bp)	Annealing temp (°C)
Genotyping				
Gpr54 floxed allele	TCCTTCTGTCATCCGGGCAT	CCTGGGCATCCTGAAGCGTT	375 (WT)<br>413 (Mut)	57
Gdf9-iCre	TCTGATGAAGTCAGGAAGAACC	GAGATGTCCTTCACTCTGATTC	No product (WT)<br>500 (Mut)	58
DNA recombination	TCCTTCTGTCATCCGGGCAT	TATCGCGGCTCAGTTCGAGG	No product (WT)<br>700 (Mut)	57
qPCR				
Gpr54	TGTGCAAATTCGTCAACTACATCC	CCCAGATGCTGAGGCTGAC	162	57
L19	CTGAAGGTCAAAGGGAATGTG	GGACAGAGTCTTGATGATCTC	195	58

Sequences of the primers and conditions used for PCR and qPCR analyses. Primers obtained from TAG Copenhagen A/S.
Mut, mutant; WT, wildtype.
