# Supplementary material for: Selective loss of kisspeptin signaling in oocytes causes progressive premature ovulatory failure
Source: Hum Reprod. 2022 Jan 17;37(4):806–21. doi: 10.1093/humrep/deab287 (PMC8971646; doi:10.1093/humrep/deab287)
Supplement: deab287_Supplementary_Table_S2 [file deab287_supplementary_table_s2.pdf]

Human Reproduction, pp. 1–1, 2022
https://doi.org/10.1093/humrep/deab287

human reproduction

SUPPLEMENTARY DATA

Supplementary Table SII List of 161 differentially expressed genes in the ovary of OoGpr54$^{-/-}$ mice displaying premature ovulatory failure.

Gene ID	Forward ROTS-statistic	*P*-value	FDR
Smoc2	7.318691	0	0
Aldh1a1	5.978873	0	0
Tmem86a	−5.49034	0	0
Serpin3c	5.398917	2.14E−07	0
Serpin5	5.301053	4.29E−07	0
Ogn	5.232464	4.29E−07	0
Anxa3	4.796188	1.93E−06	0
Kitl	4.752356	1.93E−06	0
Lyz2	4.645158	1.93E−06	0
Ndrg2	4.484367	2.57E−06	0
Trib2	4.36679	4.50E−06	0
Ctss	4.28647	5.57E−06	0
Tyrobp	4.230117	5.79E−06	0
5730469M10Rik	4.228242	6.00E−06	0
Apoe	4.138239	7.72E−06	0
Rarres2	4.112079	8.14E−06	0
Ctsd	3.998912	9.86E−06	0
Gpnmb	3.862838	1.39E−05	0
Ctsh	3.857305	1.39E−05	0
Mgst2	−3.80179	1.61E−05	0
Ctsb	3.748561	1.80E−05	0
AU021092	3.689422	2.16E−05	0
Mt3	3.66807	2.27E−05	0
Gsto1	−3.59548	2.89E−05	0
Ccdc3	3.514639	3.71E−05	0
Serpin3g	3.507705	3.75E−05	0
Col4a1	−3.4893	3.86E−05	0
Ptgr1	3.409002	4.61E−05	0
Gstm1	3.405441	4.63E−05	0
Hao2	−3.39055	4.82E−05	0
Gstm2	3.347015	5.47E−05	0
Creg1	3.28751	6.34E−05	0
Tmem176a	3.259299	6.88E−05	0
Pfn2	3.203965	7.99E−05	0
Angpt2	3.203646	7.99E−05	0
Wfdc2	−3.19649	8.21E−05	0
Fth1	3.188029	8.42E−05	0
Stc1	3.170284	8.79E−05	0
Klhl23	3.166522	8.89E−05	0
Plin2	3.161038	8.96E−05	0
Tpst2	−3.13172	9.69E−05	0
Tmem176b	3.129979	9.75E−05	0
Fabp5	3.129736	9.77E−05	0

(continued)

Human Reproduction, pp. 1–1, 2022
https://doi.org/10.1093/humrep/deab287

Supplementary Table SII Continued

Gene ID	Forward ROTS-statistic	*P*-value	FDR
Aldh6a1	3.129025	9.80E−05	0
D230025D16Rik	3.091482	0.000106	0
Rnd2	3.068349	0.00011	0
Cd68	3.056349	0.000113	0
Ltbp1	−3.0406	0.000116	0
C1qc	3.00816	0.000125	0
Clec7a	2.994703	0.000127	0
Mro	2.982443	0.000131	0
Fcer1g	2.973814	0.000133	0
Atp6v0d2	2.951889	0.00014	0
Ctsz	2.927793	0.000148	0
Col4a2	−2.92759	0.000148	0
Folr1	2.925697	0.000149	0
Trem2	2.891269	0.000163	0
Eri1	2.877544	0.00017	0
Mpeg1	2.861327	0.000177	0
Mmp12	2.85852	0.000179	0
Txnip	2.857211	0.000179	0
Sepp1	2.847104	0.000183	0
Cyba	2.836556	0.000187	0
Abcb1b	−2.82568	0.000191	0
C1qb	2.816585	0.000195	0
Wnt10b	−2.80967	0.0002	0
Sparc	−2.80391	0.000201	0
Lamp2	2.803435	0.000202	0
Sema3d	−2.80067	0.000204	0
Tulp2	2.798826	0.000204	0
Rnf130	2.783236	0.000213	0.011628
Pkdcc	2.742156	0.000234	0.011628
Smarca1	2.734638	0.000238	0.011628
Gpr128	2.731575	0.000242	0.011628
Lgals3	2.718228	0.000254	0.011628
Pld3	2.64412	0.000295	0.011628
Slc25a35	2.642153	0.000298	0.011628
Plin4	−2.64155	0.000298	0.011628
Sh2d4a	2.622127	0.000313	0.011628
Pla2g7	2.603493	0.000326	0.011628
Tpm4	−2.59754	0.000331	0.011628
Aspn	2.597014	0.000332	0.011628
Pgrmc1	2.590416	0.000339	0.011628
Ms4a7	2.579707	0.000349	0.011628
Me2	2.574636	0.000353	0.011628
Dcn	2.561268	0.000364	0.011628
Tuba1a	−2.52863	0.000397	0.017241

(continued)

Human Reproduction, pp. 1–1, 2022
https://doi.org/10.1093/humrep/deab287

Supplementary Table SII Continued

Gene ID	Forward ROTS-statistic	*P*-value	FDR
Ndfip1	2.524247	0.000402	0.018868
Tagln2	−2.50198	0.000421	0.018868
Oca2	2.488925	0.000435	0.018868
Col3a1	−2.47871	0.000447	0.018868
Shisa5	−2.47672	0.000449	0.018868
Ecm2	2.471007	0.000454	0.018868
Thra	2.462262	0.000463	0.018868
Laptm4a	2.462104	0.000463	0.018868
Fkbp5	−2.45721	0.000469	0.018868
Pcx	−2.44529	0.000484	0.018868
Ephx2	−2.44147	0.000489	0.018868
Gas6	2.423725	0.000512	0.018868
Pgap2	2.423367	0.000512	0.018868
Col6a4	2.402717	0.000537	0.018868
Sav1	−2.40233	0.000537	0.018868
Bcar3	2.397374	0.000544	0.018868
Acat1	2.388421	0.000555	0.018868
Vcam1	2.386771	0.000557	0.018868
Cd5l	2.38297	0.000562	0.018868
Bzw1	−2.37097	0.000575	0.023364
Sgk1	−2.35682	0.000598	0.026087
Lipa	2.349396	0.00061	0.026087
Gsta2	2.34142	0.000624	0.026087
Mmp14	−2.33587	0.000634	0.026087
Cfh	2.335145	0.000635	0.026087
Myl9	2.317288	0.000661	0.026087
Adck3	2.317219	0.000661	0.026087
Gm2a	−2.3122	0.00067	0.026087
Anxa2	−2.30458	0.000686	0.029915
Tmem164	2.304287	0.000686	0.029915
Lgmn	−2.30224	0.00069	0.03125
Nudt4	−2.2953	0.000703	0.03125
9930013L23Rik	−2.28039	0.000732	0.03125
Itgb2	2.273766	0.000744	0.03125
Dhrs3	2.27144	0.000747	0.03125
2610507I01Rik	2.262351	0.000763	0.03125
Unc119	2.261675	0.000764	0.03125
St3gal6	2.261076	0.000766	0.03125
Sorbs2	−2.25981	0.000767	0.03125
Fbxw7	2.25952	0.000767	0.03125
Clstn3	−2.25716	0.000772	0.03125
Hexb	2.24822	0.000789	0.036232
Gstt1	2.241905	0.000799	0.036232
Idh1	−2.24017	0.000802	0.036232

(continued)

Human Reproduction, pp. 1–1, 2022
https://doi.org/10.1093/humrep/deab287

human reproduction

SUPPLEMENTARY DATA

Supplementary Table SII Continued

Gene ID	Forward ROTS-statistic	*P*-value	FDR
Eif4e3	2.23705	0.000808	0.036232
Papss2	2.232209	0.000817	0.036232
Smpdl3b	−2.23006	0.000822	0.036232
Nrn1	−2.22041	0.000842	0.036232
4930583H14Rik	−2.21298	0.000855	0.036232
Osr2	2.210972	0.000859	0.036232
Mrap	−2.204	0.000873	0.036232
Tkt	−2.194	0.000891	0.039286
S100g	−2.19168	0.000897	0.039286
Cyp11a1	−2.18879	0.000904	0.039735
Hmgcs2	2.184254	0.000915	0.039735
Nr4a1	−2.17339	0.000937	0.039735
Ephx1	2.172083	0.000939	0.039735
Fabp3	2.161974	0.000962	0.039735
Dtx4	2.158955	0.00097	0.039735
Cpe	2.1538	0.000978	0.039735
Itm2b	2.153334	0.00098	0.039735
Ccdc8	2.140718	0.00101	0.039735
Laptm5	2.138992	0.001013	0.039735
Serpin1e	−2.13762	0.001017	0.039735
Fxyd6	2.119545	0.001064	0.045752
Cdkn1b	−2.10973	0.001091	0.045752
Cfd	−2.10332	0.001111	0.048387
Arsb	−2.10286	0.001112	0.048387
Acsbg1	2.099252	0.001122	0.049689
Cnn3	2.090713	0.001143	0.049689
Inppl1	2.088641	0.001149	0.049689
Lsr	−2.08591	0.001157	0.049689
Aldh2	2.076365	0.001183	0.049689
Pros1	2.074131	0.00119	0.049689

An excel file is provided showing the list of the 161 differentially expressed genes, identified by statistical analyses as being upregulated (47) or downregulated (114) in OoGpr54$^{-/-}$ mice, displaying ovulatory failure.
